# Titanium Sulfide Nanosheets Serve as Cascade Bioreactors for H_2_S‐Mediated Programmed Gas–Sonodynamic Cancer Therapy

**DOI:** 10.1002/advs.202201069

**Published:** 2022-08-26

**Authors:** Guangqiang Li, Huali Lei, Yuqi Yang, Xiaoyan Zhong, Fei Gong, Yuehan Gong, Yangkai Zhou, Yuqi Zhang, Haibin Shi, Zhidong Xiao, Zhiqiang Dong, Liang Cheng

**Affiliations:** ^1^ College of Biomedicine and Health College of Life Science and Technology Huazhong Agricultural University Wuhan 430070 China; ^2^ Institute of Functional Nano & Soft Materials (FUNSOM) Jiangsu Key Laboratory for Carbon‐Based Functional Materials & Devices Soochow University Suzhou 215123 China; ^3^ Brain Research Institute Research Center of Neurological Diseases Taihe Hospital Hubei University of Medicine Shiyan Hubei 442000 China; ^4^ Department of Toxicology School of Public Health Suzhou Medical College of Soochow University Suzhou 215123 China; ^5^ State Key Laboratory of Radiation Medicine and Protection Soochow University Suzhou Jiangsu 215123 China; ^6^ College of Science State Key Laboratory of Agricultural Microbiology Huazhong Agricultural University Wuhan 430070 China

**Keywords:** cascade bioreactors, gas therapy, immune modulation, sonodynamic therapy, TiS*_X_* nanosheets

## Abstract

Gas‐mediated sonodynamic therapy (SDT) has the potential to become an effective strategy to improve the therapeutic outcome and survival rate of cancer patients. Herein, titanium sulfide nanosheets (TiS*_X_* NSs) are prepared as cascade bioreactors for sequential gas–sonodynamic cancer therapy. TiS*_X_* NSs themselves as hydrogen sulfide (H_2_S) donors can burst release H_2_S gas. Following H_2_S generation, TiS*_X_* NSs are gradually degraded to become S‐defective and partly oxidized into TiO*_X_* on their surface, which endows TiS*_X_* NSs with high sonodynamic properties under ultrasound (US) irradiation. In vitro and in vivo experiments show the excellent therapeutic effects of TiS*_X_* NSs. In detail, large amounts of H_2_S gas and reactive oxygen species (ROS) can simultaneously inhibit mitochondrial respiration and ATP synthesis, leading to cancer cell apoptosis. Of note, H_2_S gas also plays important roles in modulating and activating the immune system to effectively inhibit pulmonary metastasis. Finally, the metabolizable TiS*_X_* NSs are excreted out of the body without inducing any significant long‐term toxicity. Collectively, this work establishes a cascade bioreactor of TiS*_X_* NSs with satisfactory H_2_S release ability and excellent ROS generation properties under US irradiation for programmed gas–sonodynamic cancer therapy.

## Graphical abstract

Titanium sulfide (TiS*_X_*) nanosheets synthesized via a convenient high‐temperature organic‐phase method are used as a cascade bioreactor for H_2_S‐mediated programmed gas–sonodynamic cancer therapy.

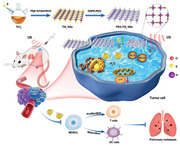

## 1 Introduction

Sonodynamic therapy (SDT), as an emerging therapeutic modality that generates reactive oxygen species (ROS) under ultrasound (US) irradiation with sonosensitizers, has been advanced treatment modality owing to its high tissue‐penetration depth, highly focused properties, and minimal damage to normal tissues.^[^ ^1^ ^]^ To date, various kinds of novel sonosensitizers, including organic and inorganic sonosensitizers, have been successfully applied for cancer SDT.^[^ ^2^ ^]^ Although multiple strategies focusing on these sonosensitizers have been widely investigated to improve the efficacy of SDT, for example, promoting electron (e^–^) conduction by establishing heterojunction construction,^[^^3]^ reducing the bandgap to accelerate electron–hole (h^+^) separation,^[^ ^4^ ^]^ and constructing defect structure to inhibit e^–^–h^+^ recombination,^[^^5]^ this field is still in its infancy due to the limited anticancer efficacy of SDT‐based monotherapy. Moreover, the complicated tumor microenvironment (TME), involving the highly reductive glutathione (GSH), hypoxia, and insufficient immune cell infiltration, further impedes the effectiveness of SDT. Thus, other treatment modalities need to be applied in combination with SDT to improve the anticancer efficacy.^[^^6]^

Gas therapy (GT), which utilizes some sorts of gases to modulate the course of diseases, has emerged as a promising strategy for cancer therapy.^[^ ^7^ ^]^ To date, various kinds of gases, including hydrogen (H_2_), nitric oxide (NO), carbon monoxide (CO), and hydrogen sulfide (H_2_S), have been reported to specifically act on the glycolysis‐based energy consumption pathway by blocking the “Warburg effect” to fight against cancer.^[7a,8^^]^ Obviously, considering the metabolic differences between the tumor cells and the normal cells, GT is highly desired and is superior to some of the clinically approved chemotherapeutics, endoradiotherapy, and even external radiotherapy, due to its high tumor cell selectivity.^[^ ^7^, ^8^ ^]^ Among them, H_2_S, as a type of gasotransmitter, plays multiple roles in cancer therapy. At higher concentrations, H_2_S not only disrupts the mitochondrial electron transport chain by inhibiting cytochrome c oxidase, but also exerts pro‐oxidant and DNA‐damaging effects.^[^ ^6^, ^9^ ^]^ Moreover, it has a positive impact on the regulation and activation of the immune system by inhibiting the accumulation of immunosuppressive myeloid‐derived suppressor cells (MDSCs) within the tumors, increasing T cells’ proliferation, and inducing the maturation of dendritic cells (DCs) to inhibit tumor metastasis.^[^ ^11^ ^]^ Based on these encouraging preliminary findings, GT has been considered as a “green” strategy to work along with SDT for enhanced long‐term anticancer effects by inhibiting the primary, metastatic, and even recurrent tumors. Recently, some studies combined SDT with GT by the mechanical combination of individual components of gas‐releasing prodrugs and sonosensitizers, which simultaneously produced gas and ROS under US irradiation, thus lacking the programmed release mechanism.^[^ ^12^ ^]^ Also, the absence of programmed generation of gas and ROS did not result in cascade‐amplified killing effects on the cancer cells. More importantly, these related studies could not modulate the TME to achieve long‐term tumor growth inhibition by gas–sonodynamic therapy alone, requiring further combination with an immune checkpoint inhibitor for immunotherapy.^[^ ^13^ ^]^ Therefore, developing a gas‐releasing sonosensitizer with programmed generation of gas as well as ROS, immunomodulation, good biosafety parameters is urgently needed for improving the efficacy of gas–sonodynamic cancer therapy.

**Scheme 1:**
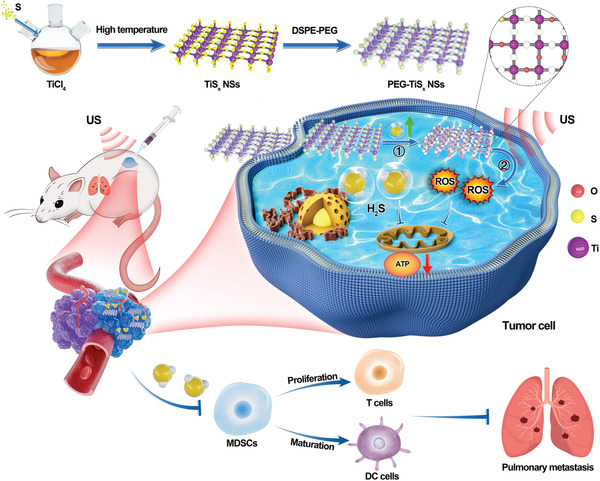
Schematic of TiS*_X_* nanosheets synthesized via a convenient high‐temperature organic‐phase method and as a cascade bioreactor for H_2_S‐mediated programmed gas–sonodynamic cancer therapy.

Transition metal dichalcogenides (TMDs), which consist of transition metals and sulfur (S) elements, are famous and well‐developed two‐dimensional (2D) nanomaterials for wide biomedical applications.^[^ ^14^ ^]^ Therefore, TMDs have the potential for H_2_S gas generation owing to the presence of S sources. Moreover, the loss of sulfur through H_2_S release in turn changes the composition and structure, which may endow defective TMDs with unique properties that can be used for cancer therapy. Herein, titanium sulfide nanosheets (TiS*_X_* NSs) were synthesized by a convenient high‐temperature organic‐phase method to serve as a cascade bioreactor for sequential gas‐enhanced SDT for cancer treatment (**Scheme** **1**). TiS*_X_* NSs themselves burst released H_2_S gas without any additional H_2_S donor. Interestingly, along with the release of H_2_S, the lack of S contributed to the formation of sulfur vacancies on the surface of TiS*_X_* NSs and formed TiO*_X_*, which significantly improved its sonodynamic performance under US irradiation. In vitro and in vivo studies confirmed the excellent effect of GT–SDT based on the cascade amplification of TiS*_X_* NSs. Importantly, the generated H_2_S activated the host's immune system to inhibit the lung metastasis of breast cancer cells and prolong the overall survival of the mice by suppressing MDSCs, promoting the expression of cytotoxic T lymphocytes, and inducing the maturation of DCs. Of note, the synthesized TiS*_X_* NSs possessed excellent biocompatibility and biodegradability, which meant that they were excreted in a timely manner. Collectively, our work highlighted that the TiS*_X_* NSs as cascade bioreactors with sequential GT and SDT performance, enhanced the host's antitumor immune response to improve the treatment efficacy, further extending the biological applications of TMDs‐based nanoplatforms.

## 2 Results and Discussion

**Figure 1:**
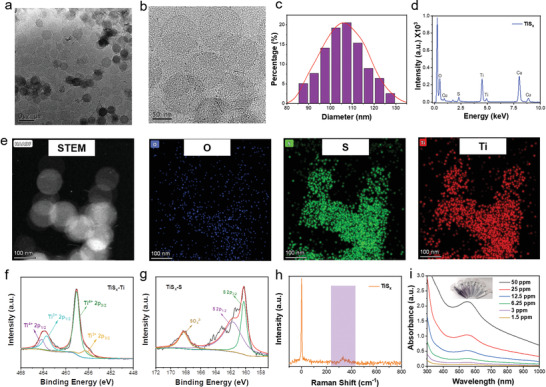
Preparation and characterization of TiS*_X_* NSs. a,b) TEM images of TiS*_X_* NSs. c) The diameter distribution of the freshly prepared TiS*_X_* NSs. d) EDX spectrum of TiS*_X_* NSs. e) Element mapping images of TiS*_X_* NSs. f,g) XPS spectra of f) Ti 2p and g) S 2p of TiS*_X_* NSs. h) Raman spectrum of TiS*_X_* NSs. i) UV–vis–NIR spectra of PEG–TiS*_X_* NSs.

The easy‐to‐obtain TiS*_X_* NSs were fabricated using the typical organic‐phase strategy with TiCl_4_ as the precursor. Transmission electron microscopy (TEM) images showed that the TiS*_X_* NSs had a uniform sheet morphology (**Figure** **1**a,b), and the diameters of the TiS*_X_* NSs were distributed between 100 and 110 nm (Figure 1c). Although the X‐ray powder diffraction (XRD) spectrum did not show any characteristic peaks of crystal structured titanium sulfide (Figure S1, Supporting Information), element mapping and X‐ray energy dispersive spectrometry (EDS) were utilized to reveal the composition of the TiS*_X_* NSs (Figure 1d,e). The existing O element in the TiS*_X_* NSs was most likely due to the partial oxidation of TiS*_X_*. The valence states of Ti and S elements were analyzed by X‐ray electron spectroscopy (XPS). The full‐scan XPS spectrum revealed typical Ti and S peaks (Figure S2, Supporting Information). In detail, for the Ti 2p region, the binding energies at 456.50, 458.07, 463.40, and 464.29 eV corresponded to Ti^3+^ 2p^3/2^, Ti^4+^ 2p^3/2^, Ti^3+^ 2p^1/2^, and Ti^4+^ 2p^1/2^, respectively (Figure 1f). In the S 2p region, the binding energies at S 2p^3/2^ and S 2p^1/2^ were obviously observed at 160.25 and 161.74 eV, respectively, revealing that the prepared TiS*_X_* NSs were rich in sulfur (Figure 1g). Raman spectrum further reflected the TiS*_X_* peaks at ≈230 and ≈330 cm^–1^, and a weak S peak was also observed at ≈320 cm^–1^ (Figure 1h; Figure S3, Supporting Information), which demonstrated that the TiS*_X_* NSs were successfully prepared. The amorphous structure was better for the subsequent H_2_S gas release.^[^ ^15^ ^]^ To enhance the biocompatibility of the TiS*_X_* NSs for biological applications, they were modified with an amphiphilic polymer, 1, 2‐distearoyl‐sn‐glycero‐3‐phosphoethanolamine‐N‐[methoxy(polyethylene glycol)] (DSPE–PEG) to form PEG–TiS*_X_* NSs through noncovalent interactions. Dynamic light scattering (DLS) revealed that the average hydrodynamic size of the PEG–TiS*_X_* NSs was ≈160 nm (Figure S4, Supporting Information), and the TEM images reflected the great dispersibility of the PEG–TiS*_X_* NSs (Figure S5, Supporting Information). The UV–vis–NIR spectra showed that the PEG–TiS*_X_* NSs possessed high NIR absorbance in a concentration‐dependent manner, enabling them to serve as contrast agents for photoacoustic imaging (Figure 1i). In addition, the PEG–TiS*_X_* NSs dispersed well in various simulated body fluids, including H_2_O, phosphate buffered solution (PBS), 0.9% NaCl, and Roswell Park Memorial Institute (RPMI) (Figure S6, Supporting Information). Combined with the scanning of UV–vis–NIR spectra, the PEG–TiS_X_ NSs were gradually degraded (Figures S7 and S8, Supporting Information), showing a great potential for biomedical applications.

**Figure 2:**
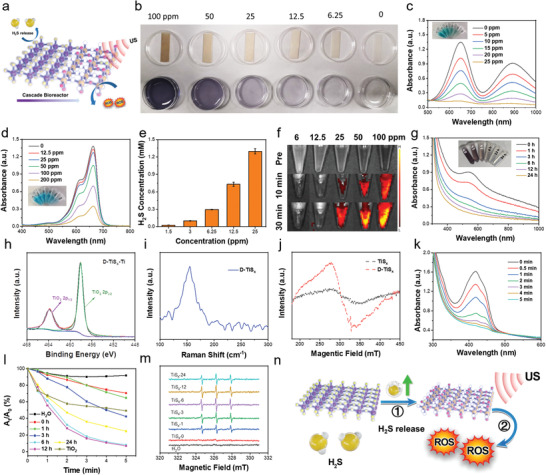
H_2_S release and sonodynamic performance of PEG–TiS*_X_* NSs. a) Schematic illustration of H_2_S release and sonodynamic properties of PEG–TiS*_X_* NSs. b–d) H_2_S release performance of PEG–TiS*_X_* NSs with different concentrations using b) lead acetate test paper, c) TMB, and d) MB as the indicators, respectively. e) H_2_S release property of PEG–TiS*_X_* NSs with different concentrations using WSP‐1 probe. f) The fluorescence imaging of PEG–TiS*_X_* NSs with different concentrations using H_2_S probe at different time points. g) UV–vis–NIR spectra of PEG–TiS*_X_* NSs with different degradation times. h) XPS spectrum of Ti 2p of D‐TiS*_X_* NSs. i) Raman spectrum of D‐TiS*_X_* NSs. j) ESR spectra demonstrating the sulfur vacancies of TiS*_X_* and D‐TiS*_X_* NSs. k) ROS generation ability of PEG–TiS*_X_* NSs after degradation for 12 h. l) Comparison of sonodynamic performance of PEG–TiS*_X_* NSs with different degradation times and commercial TiO_2_. m) ESR spectra exhibiting ^1^O_2_ generation by H_2_O and PEG–TiS*_X_* NSs with different degradation times under US irradiation. n) The schematic mechanism of TiS*_X_* NSs for sonodynamic property.

The TiS*_X_* NSs containing abundant sulfur content could be functionalized as a source of S to generate H_2_S for GT (**Figure** **2**a). To investigate the ability to release H_2_S gas, the lead acetate test paper was used as an indicator that could capture H_2_S to form black lead sulfide (PbS). The deeper color of the solution qualitatively reflected the higher concentration of the generated H_2_S (Figure 2b). In addition, the blue indigo color of oxTMB, which was the oxidized form of 3,3′,5,5′‐tetramethylbenzidine (TMB), was converted to colorless TMB under the reduction of H_2_S (Figure 2c). Similarly, the UV–vis–NIR spectra showed that the methylene blue (MB) probe further exhibited H_2_S gas generation ability with a decreasing characteristic absorption peak in a concentration‐dependent manner (Figure 2d). Washington State Probe‐1 (WSP‐1) was further applied to quantitatively detect H_2_S gas generation. It was found that the low concentration of the PEG–TiS*_X_* NSs generated enough H_2_S gas, leading to excellent efficacy of gas therapy (Figure 2e; Figures S12 and S13, Supporting Information). In addition, a reported H_2_S probe was further used to detect the H_2_S gas release, and the fluorescence signal increased gradually with the concentration and time (Figure 2f; Figure S15, Supporting Information), indicating the large amount of H_2_S release from the TiS*_X_* NSs.^[^ ^16^ ^]^ Next, the H_2_S generation ability of the differently degraded PEG–TiS*_X_* NSs was measured. The longer the degradation time was, the more the H_2_S gas was generated by PEG–TiS*_X_* (Figure S9, Supporting Information). After the different degradation time, the other TMB and MB probes were used to detect reduced H_2_S gas release (Figures S10 and S11, Supporting Information), which was further precisely quantified by WSP‐1 detection (Figure S14, Supporting Information). In addition, it possessed the property of pH‐dependent H_2_S release under weakly acidic conditions (Figure S16, Supporting Information).

Interestingly, the TiS*_X_* NSs were gradually degraded upon H_2_S release. To investigate the changes in the morphology and structure of TiS*_X_* NSs, a series of different characterization approaches were used. With a long degradation time, the color of the PEG–TiS*_X_* NSs changed from atropurpureus to nearly white and the UV–vis–NIR spectra gradually decreased (Figure 2g). However, the DLS and TEM images showed little change of its size (Figures S17 and S18, Supporting Information), and there was no significant change in the structure as observed by XRD analysis (Figure S19, Supporting Information). The EDS spectrum revealed that the content of S was reduced, while the O content was enhanced (Figure S20, Supporting Information), and the element mapping images of D‐TiS*_X_* showed similar results (Figure S21, Supporting Information). XPS spectra revealed that the TiO_2_ peak appeared (Figure 2h), and owing to H_2_S gas release, the S signal became weak (Figure S22, Supporting Information). All the above results revealed that the TiS*_X_* NSs were partially oxidized to TiO*_X_* upon H_2_S gas release. Furthermore, Raman spectrum showed a TiO_2_ signal peak at ≈155 cm^–1^, which further confirmed the formation of TiO*_X_* (Figure 2i). Notably, the electron spin resonance (ESR) spectrum showed the signature of sulfur vacancies at *g* = 2.002. The ESR signal increased to 4.5 times with high degradation (Figure 2j; Figure S23, Supporting Information), which could be ascribed to the free electrons generated by the sulfur vacancies of TiS*_X_*.^[^ ^17^ ^]^ To further verify its vacancy, the photoluminescence (PL) spectra were measured.^[^ ^18^ ^]^ It could be seen that the PL spectra of the PEG–D‐TiS*_X_* NSs were much lower than that of the PEG–TiS*_X_* NSs, potentially due to the fact that most of the vacancies in the PEG–D‐TiS*_X_* NSs captured the photoexcited electrons, and then the decreased excitation energy reduced the photoluminescence intensity (Figure S24, Supporting Information).

It has been reported that the defective structure could capture the e^–^ to enhance ROS generation.^[^ ^4^, ^18^ ^]^ Therefore, we hoped that the formation of TiO*_X_* and S defects might improve the sonodynamic performance of D‐TiS*_X_* NSs. Afterward, the sonodynamic efficacies of the TiS*_X_* NSs with different degradation degrees were evaluated using the 1,2‐diphenylisobenzofuran (DPBF) probe. From the UV–vis spectra, the attenuation rates of DPBF by different degraded TiS*_X_* NSs were calculated (Figure 2l). After 12 h of degradation, the D‐TiS*_X_* NSs produced the maximum ROS under US irradiation (Figure 2k). The commercial sonosensitizer TiO_2_ was also detected as the positive control, and the ROS generation efficacy of D‐TiS*_X_* NSs was much higher. However, the ROS generation ability of the D‐TiS*_X_* NSs after degradation for 24 h declined, potentially due to the decomposition of the TiS*_X_* NSs (Figure S25, Supporting Information). Meanwhile, the TiS*_X_* NSs themselves, with various degradation time, hardly generated ROS without US irradiation (Figure S26, Supporting Information). Furthermore, similar degree of ROS generation was found by using the diphenylamine (DPA) probe (Figures S27 and S28, Supporting Information). ESR spectra further distinguished the type of ROS, wherein the typical characteristic peaks of 1:1:1 appeared, reflecting ^1^O_2_ generation under US irradiation (Figure 2m; Figure S34, Supporting Information). In addition, TMB and *o*‐phenylenediamine (OPD) probes were used to detect other types of ROS, and it was found that there was no obvious signal change. The ESR spectra revealed that there were no hydroxyl radical (·OH) peaks, which indicated that the PEG–TiS*_X_* NSs generated ^1^O_2_ rather than ·OH under US irradiation (Figures S29–S31, Supporting Information). Significantly, the D‐TiS*_X_* NSs showed excellent stability under US irradiation (Figures S32 and S33, Supporting Information). TiO*_X_* is a known sonosensitizer, and the vacancies capture electrons (e^–^) to inhibit the recombination of e^–^ and hole (h^+^) pairs. According to the above results, the potential mechanism was proposed (Figure 2n). With the release of H_2_S gas, S vacancies were formed and increased; meanwhile, partial S elements were replaced by O to form TiO*_X_* on the surface of TiS*_X_* NSs. The formation of TiO*_X_* endowed the sonodynamic effect, and the S vacancies further captured the e^–^, and then combined with O_2_ to produce ^1^O_2_ under US irradiation. Therefore, the cascade D‐TiS*_X_* NSs served as efficient sonosensitizers for SDT of cancer.

**Figure 3:**
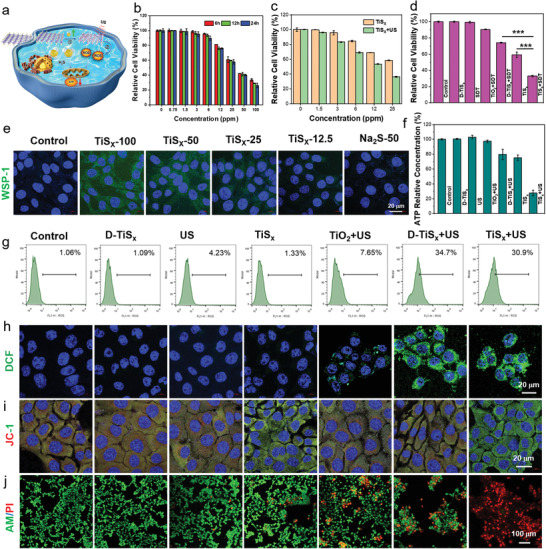
In vitro H_2_S release and SDT properties of PEG–TiS*_X_* NSs. a) Schematic illustration of PEG–TiS*_X_* NSs for H_2_S release‐mediated GT and SDT in vitro. b) Relative cell viability of 4T1 cells after treatments with PEG–TiS*_X_* NSs of different degradation time. c) Relative cell viability of 4T1 cells after incubation with different concentrations of PEG–TiS*_X_* NSs or combined with US (30 kHz, 5 min) irradiation for 12 h. d) Relative cell viability of 4T1 cells after different treatments. e) Performance of H_2_S release in vitro with PEG–TiS*_X_* NSs of different concentrations by the WSP‐1 probe. f) The relative concentration of intracellular ATP after different treatments. g) Flow cytometry to quantificationally display intracellular ROS generation after different treatments. h–j) Confocal images of 4T1 cells stained with h) DCFH‐DA, i) JC‐1, and j) Calcein acetoxymethyl ester/propidiumiodide (Calcein AM/PI) after various treatments, respectively. Data are presented as mean values ± standard deviation (SD, *n* = 6).

Motivated by the outstanding H_2_S release and sonodynamic performance of the TiS*_X_* NSs, we further evaluated its in vitro anticancer effects (**Figure** **3**a). First, the standard methyl thiazolyl tetrazolium (MTT) assay was performed to evaluate the cytotoxicity of PEG–TiS*_X_* NSs. Owing to H_2_S release, the PEG–TiS*_X_* NSs exhibited significant lethality toward 4T1 cells (Figure 3b). Interestingly, the human umbilical vein endothelial cells (HUVECs) displayed higher viability than 4T1 cells after treatment with the PEG–TiS*_X_* NSs (Figure S35, Supporting Information). The weakly acidic condition of 4T1 cells constantly caused H_2_S release, inducing more severe damage. The MTT assay results for PEG–D‐TiS*_X_* NSs demonstrated that it was biocompatible (Figure S36, Supporting Information). Next, the standard MTT assay showed that SDT further killed the cancer cells and improved the therapeutic efficacy (Figure 3c). Meanwhile, comparing the overall therapeutic efficacy, the relative cell viabilities of the D‐TiS*_X_* +US and TiO_2_ +US groups were ≈74.15% and ≈90.52%, respectively. However, the viability of the TiS*_X_*‐treated group was reduced to ≈50.18%. Interestingly, the cell viability of the TiS*_X_* +US group was further reduced to ≈33.06% (Figure 3d), which suggested that H_2_S‐mediated GT combined with SDT induced greater cytotoxic effects than the single treatment. Subsequently, the WSP‐1 probe with green fluorescence showed that H_2_S was generated within the cells, and the content of H_2_S from TiS*_X_* NSs was much higher than that of the standard donor of Na_2_S (Figure 3e; Figure S37, Supporting Information).

To further demonstrate the sonodynamic performance of TiS*_X_* NSs, the intracellular ROS level was evaluated by the 2,7‐dichlorofluorenscein diacetate (DCFH‐DA) probe. TiO_2_ only induced ≈7.65% ROS upon US irradiation, while high‐intensity ROS was induced in the D‐TiS*_X_* +US groups (≈34.9%) and the TiS*_X_* +US groups (≈30.9%), respectively (Figure 3g). Similarly, strong green fluorescence was observed in the D‐TiS*_X_* +US and TiS*_X_* +US groups, which reflected the abundant generation of ROS by the D‐TiS*_X_* NSs upon US irradiation to kill cells (Figure 3h; Figure S38, Supporting Information). Afterward, the mechanism of action and the therapeutic efficacy were investigated. According to previous studies, H_2_S‐mediated inhibition of mitochondrial respiration and ATP generation have been reported to promote cell necrosis and apoptosis.^[^ ^7c^ ^]^ Consequently, the JC‐1 kit was used to determine the mitochondrial membrane potential to verify the induction of apoptosis. Stronger green fluorescence was observed in the TiS*_X_* and TiS*_X_* +US groups, because both H_2_S and ROS generation could impair mitochondrial respiration (Figure 3i; Figure S39, Supporting Information). In addition, the ATP assay kit showed that excessive H_2_S and abundant ROS from SDT together inhibited ATP generation and then induced apoptosis (Figure 3f). The live/dead co‐staining assay ultimately confirmed the antitumor effects, with the TiS*_X_* +US group inducing the highest cytotoxicity to 4T1 cells (Figure 3j). In conclusion, the cascade bioreaction of TiS*_X_* NSs showed outstanding H_2_S release ability and subsequently enhanced the SDT performance to inhibit mitochondrial respiration and ATP synthesis to further kill the cancer cells.

**Figure 4:**
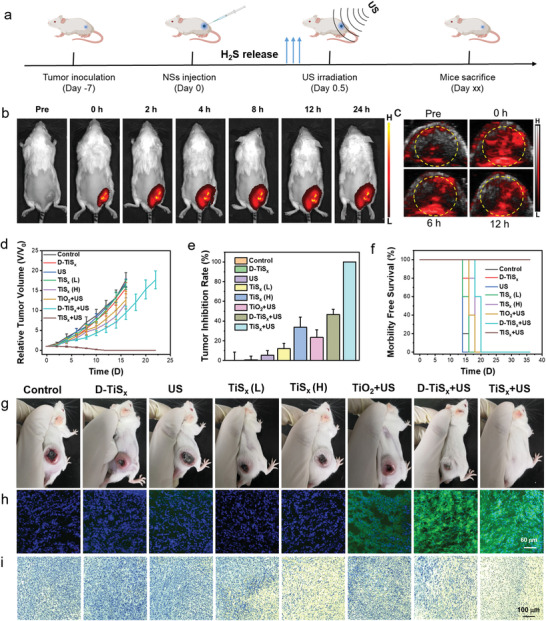
In vivo H_2_S release mediated GT and SDT properties. a) The therapeutic schedule for 4T1 tumor‐bearing mice. b) The fluorescence imaging of the retention after intratumoral (i.t.) injection with Cy5.5‐labeled PEG–TiS*_X_* NSs. c) The PA imaging of the PEG–TiS*_X_* NSs at different time points. d) The relative tumor volume in mice with various treatments. e) The tumor inhibition rates of mice with different management strategies. f) The overall survival curves of mice with different treatments. g) Photograph showing the tumor volume of mice after various treatments on the 16^th^ day. h,i) Tumor staining for h) ROS and i) TUNEL after different treatments. Data are presented as mean values ± SD (*n* = 5 biologically independent mice).

Encouraged by the excellent H_2_S release and sonodynamic performance, we further investigated the in vivo antitumor effects of GT and SDT by the PEG–TiS*_X_* NSs (**Figure** **4**a). First, the Cy5.5‐labeled PEG–TiS*_X_* NSs showed long‐term retention in the tumor upon intratumoral (i.t.) injection (Figure 4b; Figures S40 and S41, Supporting Information). Then, the photoacoustic (PA) signal gradually reduced, and the signal tended to reach that of the control group's level at 12 h (Figure 4c; Figure S42, Supporting Information), indicating that most of the H_2_S was released. Next, BALB/c mice bearing 4T1 tumors were randomly divided into eight groups: 1) control; 2) D‐TiS*_X_* (5 mg kg^−1^); 3) US; 4) TiS*_X_*‐2 mg kg^−1^ (TiS*_X_*‐(L)); 5) TiS*_X_*‐5 mg kg^−1^ (TiS*_X_*‐(H)); 6) TiO_2_ +US (5 mg kg^−1^); 7) D‐TiS*_X_* +US (5 mg kg^−1^); and 8) TiS*_X_* +US (5 mg kg^−1^). The PEG–TiS*_X_* NSs were administered intratumorally. Based on the in vitro degradation kinetics of the PEG–TiS*_X_* NSs and the result of PA signal in vivo, the tumors were exposed to US irradiation after 12 h of the i.t. injection. It was obvious that the tumor growth in the TiS*_X_* +US group was completely inhibited, and tumors in the TiS*_X_* (H) and D‐TiS*_X_* +US groups grew slowly, reflecting the high efficacy of the treatments in delaying tumor growth (Figure 4d). Photographs of tumors after treatments for 6 and 16 days showed similar results, respectively (Figure 4g; Figure S43, Supporting Information). From the survival analysis, the TiS*_X_* +US group exhibited the best therapeutic effect, with an inhibitory rate of 100%. However, the TiS*_X_* (H) and D‐TiS*_X_* +US groups showed only ≈33.9% and ≈46.7% tumor growth inhibition, respectively (Figure 4e). Moreover, all the mice in the TiS*_X_* +US group survived without any tumor recurrence, but the tumors in the other control groups gradually reached the death criteria, indicating the excellent therapeutic effects with programmed H_2_S release and US‐triggered ROS generation (Figure 4f).

The underlying tumoricidal mechanism of the PEG–TiS*_X_* NSs was further explored. First of all, the in vivo H_2_S release was detected by the fluorescence imaging of the reported ZM106‐NB probe, and it was found that the H_2_S release was about two times higher in the TiS*_X_* group than the control group (Figure S44, Supporting Information), demonstrating the high H_2_S release ability of the TiS*_X_* NSs. Next, we conducted ROS staining of tumors after the different treatments with a DCFH‐DA probe. Stronger green fluorescence appeared in the D‐TiS*_X_* +US and TiS*_X_* +US groups, while weaker signals appeared in the TiO_2_ +US and the control groups (Figure 4h; Figure S45, Supporting Information). Furthermore, the blood oxygen saturation levels measured by PA imaging reflected that the released H_2_S and the generated ROS under US irradiation inhibited mitochondrial respiration and ATP generation that further induced the cancer cell death (Figure S46, Supporting Information). Subsequently, the immunohistochemical TdT‐mediated dUTP Nick‐end labeling (TUNEL) analysis also reflected the maximum damage of tumor cells in the TiS*_X_* +US group, with nearly all the tumor cells being severely destroyed. However, the tumor cells in the TiS*_X_* (H), TiO_2_ +US, and D‐TiS*_X_* +US groups were partly damaged (Figure 4i). Besides, the hematoxylin and eosin (H&E) staining showed similar results (Figure S47, Supporting Information). Overall, the TiS*_X_* NSs possessed outstanding in vivo therapeutic effects with H_2_S release, followed by ROS generation upon US irradiation.

**Figure 5:**
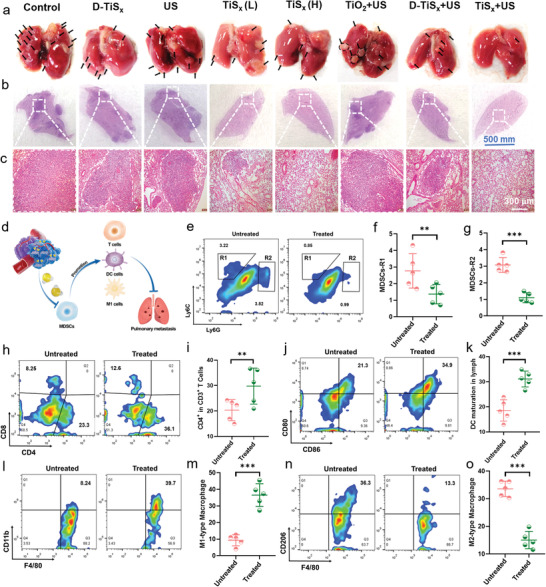
The immunoreaction regulation effect of H_2_S. a–c) Images of lung metastases in different groups by a) digital photography and b,c) the corresponding H&E staining of lung tissues on the 35^th^ day. d) Schematic illustration of H_2_S‐regulated immunoreaction. e–i) Flow cytometry plots and related quantification of e–g) MDSCs and h,i) CD4^+^ T cells in tumors treated with PEG–TiS*_X_* NSs. j) Flow cytometry plots and k) related quantification of mature DCs in lymph. l–o) Flow cytometry plots and related quantification of l,m) M1 cells and n,o) M2 cells in tumors treated with PEG–TiS*_X_* NSs. Data are presented as mean values ± SD (*n* = 5 biologically independent mice).

During cancer treatment, the mice in the control group gradually died, while the TiS*_X_*‐treated mice still survived for a long time, this difference was potentially due to the occurrence of highly visible metastases in the control group (**Figure** **5**a). Furthermore, H&E staining was utilized to visualize the metastases within the lungs, and fewer metastatic nodules were observed in the TiS*_X_* and TiS*_X_* +US treated groups (Figure 5b,c). To determine whether the TiS*_X_* NSs had the ability to inhibit lung metastasis, the released H_2_S‐mediated immunoregulatory effect was further investigated (Figure 5d). We first induced the MDSCs by adding granulocyte‐macrophage colony stimulating factor (GM‐CSF) and interleukin 6 (IL‐6) to the bone marrow precursor cells, and then treated them with PEG–TiS*_X_* NSs.^[^ ^20^ ^]^ The results showed that the released H_2_S greatly inhibited the expression of MDSCs (Figure S48, Supporting Information). Next, the tumors and the nearby lymph nodes were collected to analyze different immune cells on day 7 after treatment. The TiS*_X_* NSs inhibited MDSCs’ expansion in the tumors, and both monocytic (mo, R1) and granulocytic (gr, R2) cells were inhibited by the released H_2_S gas (Figure 5e–g). The suppression of MDSCs might have activated the proliferation of cytotoxic T cells. Therefore, we further evaluated the T cells, and the results reflected that the CD4^+^ T cells’ infiltration increased in the tumor, which indirectly showed that lots of antibody could have been produced to suppress tumor growth (Figure 5h,i). DCs are a type of antigen‐presenting cells (APCs) that play a significant role during the immune response. Therefore, the treatment of TiS*_X_* NSs promoted DCs maturation in nearby tumor lymph nodes (Figure 5j,k). In addition, macrophages, known as a kind of phagocyte, were also analyzed for the two main phenotypes: classically activated (M1) and alternatively activated (M2) macrophages. M1 cells are known to release pro‐inflammatory cytokines, such as IL‐6, interleukin 12 (IL‐12), and tumor necrosis factor *α* (TNF‐*α*), to induce cancer cell apoptosis.^[^ ^21^ ^]^ Interestingly, TiS*_X_* NSs’ treatment promoted M2 to M1 polarization (Figure 5l–o), and the related cytokines were all increased in the tumors (Figure S49, Supporting Information). In summary, TiS*_X_* NSs, as a cascade bioreactor, activated the immune system and greatly inhibited pulmonary metastasis of 4T1 breast tumors and prolonged the survival time of mice.

Finally, the biosafety of inorganic nanomaterials must be considered for their wide biomedical applications. First, H&E staining of the vital organs (heart, liver, spleen, lung, kidney, and brain) was performed, and the results showed no obvious morphological changes with different durations of exposure to the TiS*_X_* NSs (Figure S50, Supporting Information). The body weight increased in all the groups, indicating that the TiS*_X_* NSs had no evident systemic toxicity at the dose of 10 mg kg^−1^ (Figure S51, Supporting Information). Afterward, the biodistribution and metabolism of TiS*_X_* NSs were investigated. The biodistribution profile indicated that the TiS*_X_* NSs mainly accumulated in organs that were rich in blood supply, such as the liver and spleen, and the content gradually decreased with time. To verify the metabolic route, the feces and urine of mice were collected to detect the Ti content. It was found that the TiS*_X_* NSs were mainly excreted out of the body through the feces, and ≈71.34 µg of Ti was detected within 7 days, accounting for 1/3 of the injected dose (Figure S52, Supporting Information). TEM images revealed a mixture of nanosheets and nanodots of PEG–TiS*_X_* NSs with amorphous structure after its degradation under physiological condition at 14 days (Figure S53, Supporting Information), indicating that the TiS*_X_* NSs were excreted through hepatic metabolism. Finally, the blood routine and blood biochemical tests showed that the hematological indexes were similar across all the experimental groups (Figure S54, Supporting Information). In short, the TiS*_X_* NSs possessed good biosafety for biological applications.

## 3 Conclusions

In summary, TiS*_X_* NSs were successfully established as cascade bioreactors to achieve programmed cancer therapy through H_2_S release‐mediated GT–SDT under US irradiation. A convenient method of organic‐phase synthesis was applied to fabricate the multifunctional bioreactor, TiS*_X_* NSs. Following modification with PEG, the PEG–TiS*_X_* NSs exhibited great biocompatibility. The TiS*_X_* NSs burst released vast amounts of H_2_S gas owing to the abundant sulfur element in the samples. With the release of H_2_S, the TiS*_X_* NSs degraded and were gradually oxidized, forming sulfur vacancies on the D‐TiS*_X_* NSs, which made the D‐TiS*_X_* NSs a highly efficient sonosensitizer with excellent sonodynamic activities. The cascade bioreactor of TiS*_X_* NSs with superb H_2_S release ability and sonodynamic effects significantly inhibited mitochondrial respiration and ATP synthesis, promoting therapeutic efficacy against cancer cells through apoptosis induction both in vitro and in vivo. In addition, H_2_S gas played a positive role in activating the immune system to effectively inhibit the pulmonary metastasis of breast cancer cells by inhibiting MDSCs, inducing T cells’ proliferation and DCs’ maturation. Importantly, TiS*_X_* NSs were excreted out of the body without any obvious long‐term toxicity. In conclusion, the current work established a cascade amplifying TiS*_X_* NSs for programmed cancer gas–sonodynamic therapy, extending the biomedical applications of TMDs‐based nanoplatforms for highly efficient cancer therapy by suppressing the primary tumor growth and inhibiting metastatic spread to other organs.

## Conflict of Interest

The authors declare no conflict of interest.

## Supporting information

Supporting Information

## Data Availability

The data that support the findings of this study are available from the corresponding author upon reasonable request.
