# Supplementary material for: Titanium Sulfide Nanosheets Serve as Cascade Bioreactors for H2S‐Mediated Programmed Gas–Sonodynamic Cancer Therapy
Source: Adv Sci (Weinh). 2022 Aug 26;9(30):2201069. doi: 10.1002/advs.202201069 (PMC9596849; doi:10.1002/advs.202201069)
Supplement: Supplementary file 1 — Supporting Information [file ADVS-9-2201069-s001.pdf]

ADVANCED SCIENCE
Open Access

Supporting Information

for *Adv. Sci.*, DOI 10.1002/advs.202201069

Titanium Sulfide Nanosheets Serve as Cascade Bioreactors for H_2S-Mediated Programmed Gas–Sonodynamic Cancer Therapy

Guangqiang Li, Huali Lei, Yuqi Yang, Xiaoyan Zhong, Fei Gong, Yuehan Gong, Yangkai Zhou, Yuqi Zhang, Haibin Shi, Zhidong Xiao, Zhiqiang Dong* and Liang Cheng**

Supporting Information

Titanium Sulfide Nanosheets Serve as Cascade Bioreactors for H_2S-mediated Programmed Gas-Sonodynamic Cancer Therapy

Guangqiang Li[1,2,3], Huali Lei[2], Yuqi Yang[2], Xiaoyan Zhong[4,], Fei Gong[2], Yuehan Gong[2], Yangkai Zhou[2], Yuqi Zhang[5], Haibin Shi[5], Zhidong Xiao[6], Zhiqiang Dong[1,3,*], Liang Cheng[2,*]*

[1]College of Biomedicine and Health, College of Life Science and Technology, Huazhong Agricultural University, Wuhan, 430070, China

[2]Institute of Functional Nano & Soft Materials (FUNSOM), Jiangsu Key Laboratory for Carbon-Based Functional Materials & Devices, Soochow University, Suzhou, 215123, China

[3]Brain Research Institute, Research Center of Neurological Diseases, Taihe Hospital, Hubei University of Medicine, Shiyan, Hubei, 442000, China

[4]Department of Toxicology, School of Public Health, Suzhou Medical College of Soochow University, Suzhou, 215123, China

[5]State Key Laboratory of Radiation Medicine and Protection, Soochow University, Suzhou, Jiangsu, 215123, China

[6]College of Science, State Key Laboratory of Agricultural Microbiology, Huazhong Agricultural University, Wuhan, 430070, China

*E-mails: lcheng2@suda.edu.cn; dongz@mail.hzau.edu.cn; xyzhong@suda.edu.cn

1. Experimental section

Materials

Titanium tetrachloride ($TiCl_4$), Methylene chloride, hexamethylene, and anhydrous ethyl alcohol were purchased from Sinopharm Chemical Regent Co., Ltd. Sulfur powder (S), oleylamine (OM), 1-octadecene (ODE), 1,2-diphenylisobenzofuran (DPBF), 5,5-dimethyl-pyrroline-N-oxide (DMPO) and 2,2,6,6-Tetramethyylpiperidine (TEMP) were obtained from Sigma-Aldrich. 3,3,5,5-tetramethylbenzidine (TMB), and methylene blue (MB) were purchased from J&K Chemical Co., Ltd. Commercial TiO_2 nanoparticles (NPs) was purchased from Macklin Chemical Co., Ltd, and DSPE-PEG (MW 2000) was obtained from Pengsheng Biotechnology Co., Ltd. JC-1 kit and ATP assay kit were got by Beyotime Co., Ltd. Washington state probe -1 (WSP-1) was purchased from Maokang biological Co., Ltd. All chemical reagents were analytical grade and used without further purification.

Synthesis of TiS_X nanosheets

TiS_X nanosheets (TiS_X NSs) were synthesized by a high-temperature organic-phase method. Firstly, 20 mL of OM and 10 mL of ODE were mixed in a three-necked flask under vigorous magnetic stirring. The mixture was heated to 120 ^oC, and then, 440 μL of $TiCl_4$ was added and maintained at 120 ^oC for 30 mins with nitrogen protection. Next, the mixture was further heated to 260 ^oC and 256 mg of S in 4 mL of OM was slowly injected into the solution. Finally, the reaction was maintained at 260 ^oC for 10 mins under nitrogen protection. After that, the product was naturally cooled down to room temperature and collected by washing with hexamethylene and anhydrous ethyl alcohol.

The TiS_X NSs were modified with DSPE-PEG for biomedical applications. Briefly, 20 mg of TiS_X NSs and 60 mg of DSPE-PEG were mixed in 6 mL of dichloromethane under ultrasonication for 10 mins, followed by removing dichloromethane through a rotary evaporator. The PEG-TiS_X NSs were obtained and re-dispersed in deionized water and stored at 4 ^oC for future use.

Characterization

The morphologies of TiS_X NSs were characterized by transmission electron microscope (TEM, tecnai F20). The crystal structure and surface chemical composition of TiS_X NSs were measured by

X-ray diffraction (XRD, Panalytical Empyrean) and X-ray photoelectron spectroscopy (XPS, ESCALab 250Xi). The absorption spectra were obtained by UV-vis-NIR spectrophotometer (GenesysTM 10S UV-Vis, Thermo Scientific). Singlet oxygen ($^1O_2$) and sulfur vacancy were detected by electron spin resonance (ESR) spectrometer (Bruker EMXplus). The Hainertec (SuZhou) Co., Ltd. Offered the ultrasonic generator. The absolute concentration of Ti ions was measured by inductively coupled plasma-optical emission spectroscopy (ICP-OES, Avio 200).

Hydrogen sulfide (H_2S) release of TiS_X-PEG NSs

The H_2S generation was qualitatively analyzed by TMB and MB probes, respectively. Firstly, 200 μL of $FeCl_2$ (1 mg/mL) and 200 μL of H_2O_2 (10 mM) were added to 10 mL of deionized water. Next, 50 μL of TMB (0.5 mM) was added to the above solution to form blue oxTMB by ·OH oxidation. Finally, the PEG-TiS_X NSs with different degradation time at various concentrations were added to the above solution, then the declined absorbance of TMB at 655 nm reflected the release of H_2S by PEG-TiS_X NSs, meaning that H_2S could reverse oxTMB to colorless TMB. Similarly, the MB probe was with the same principle to reveal the H_2S generation. Furthermore, H_2S release was quantitatively analyzed by WSP-1 probe. In short, 50 μL of PEG-TiS_X NSs with different concentrations were incubated with 50 μL of WSP-1 probe (50 μM) for 1 h at 37 ^oC, followed by measuring the fluorescence intensity in Microplate Reader (Ex = 465 nm, Em = 515 nm).

ROS generation of PEG-TiS_x NSs by US activation

The DPBF was typically used as a molecular probe to detect the production of ROS. 1 mL of PEG-TiS_X NSs (25 μg/mL based on Ti) was mixed with 20 μL of DPBF (1 mg/mL in ethanol solution). The mixture was irradiated with US (30 kHz, 3W/cm^2) for different time in the dark. The declined absorbance of DPBF at 420 nm reflected the generation of ROS quantificationally by PEG-TiS_X NSs.

To distinguish the types of ROS, TEMP as the trapping agent of $^1O_2$ and DMPO as the trapping agent of hydroxyl radical (·OH) were used to conduct ESR measurement. 20 μL of TEMP or DMPO was added into 1 mL of PEG-TiS_X NSs (15 μg/mL) and irradiated by US (30 kHz, 3 W/cm^2)

for 2 mins. The ESR spectrometer displayed the characteristic peak of $^1O_2$.

Cellular experiments

The 4T1 murine breast cancer cell line was obtained from American Type Culture Collection (ATCC) and cultured in the standard cell culture medium at the condition of 37 °C, 5% CO_2. For the cytotoxicity test *in vitro*, the standard 3-(4,5-dimethyl-2-thiazolyl)-2,5-diphenyl-2-Htetrazolium bromide (MTT) assay was conducted. The different concentrations of PEG-TiS_X NSs (0-50 ppm), PEG-D-TiS_X NSs (0-100 ppm) were added into the 96-well plates with 4T1 cell, and incubated for various time (6, 12, and 24 h), and the different concentrations of PEG-TiS_X NSs (0-50 ppm) were added into the 96-well plates with HUVECs for 12 h. For the study of gas therapy (GT) and sonodynamic therapy (SDT), 4T1 cells were incubated with 25 ppm of PEG-TiS_X NSs for 12 h, and then under the US irradiation (30 kHz, 3 W/cm^2, 1 min per cycle, 5 cycles). The relative cell viabilities were measured by MTT assay.

For the H_2S detection, 4T1 cells were incubated with the PEG-TiS_X NSs in different concentrations for 6 h, and the WSP-1 probe (50 μM) was added to react for 1 h.

For the ATP detection, 4T1 cells were incubated with PEG-TiS_X NSs for 12 h. After the different treatments, the cells were collected, and washed three times. Next, the cell members were broken to detect ATP level with the diagnostic kit.

For the live/dead staining, 4T1 cells were divided into seven groups: (1) Control, (2) D-TiS_X-NSs, (3) TiS_X NSs, (4) US, (5) TiO_2 NSs+ US, (6) D-TiS_X NSs +US, and (7) TiS_X NSs +US. 4T1 cells were incubated with PEG-TiS_X NSs for 12 h. After the different treatments, the Calcein AM (AM) and propidium iodide (PI) dyes were used to stain live and dead cells, respectively. For ROS staining, 4T1 cells after different treatments were incubated with the DCFH-DA (20 μM) probe for 30 mins.

For MDSCs cultured, the MDSCs *in vitro* differentiation was performed as previously described.[1] Briefly, bone marrow cells were isolated and cultured for 4 days in 10 mL RPMI-1640 with 10% FBS, 1% penicillin/ streptomycin, 10ng/mL GM-CSF and 10ng/mL IL-6.

Tumor model

Balb/c mice were purchased from Nanjing Sikerui Biological Technology Co., Ltd, and all the

animal experiments were carried out under the permission by Laboratory Animal Center of Soochow University.

The retention and degradation of PEG-TiSx *in vivo*

The cy5.5 labelled PEG-TiSx NSs (2mg/Kg) were intratumorally (i.t.) injected into the tumor, and the fluorescence signals were observed in the different time. The PEG-TiSx NSs were i.t. injected into the tumor, and the PA signals were detected at 800 nm in the different.

Programmed GT and SDT by cascade bioreactor of PEG-TiSx *in vivo*

The mice bearing 4T1 tumor (~100 mm^3) were randomly divided into eight groups (n=5 per group): (1) Control, (2) D-TiS_X NSs (i.t. injection, 5 mg/kg), (3) US; (4) TiS_X NSs (L) (i.t. injection, 2 mg/kg), (5) TiS_X NSs (H) (i.t. injection, 5 mg/kg), (6) TiO_2 NSs (i.t. injection, 5 mg/kg) +US, (7) D-TiS_X NSs (i.t. injection, 5 mg/kg) +US, and (8) TiS_X NSs (i.t. injection, 5 mg/kg) +US. 12 h after i.t. injection, the tumors were treated with US irradiation (30 kHz, 3 W/cm^2, 1 min per cycle, 15 cycles). The tumor volume and body weight were recorded every two days. The tumor volume was calculated by the formula: Tumor volume (mm^3) = $ab^2/2$, where a was the max length (mm) of the tumor, and b was the min-width (mm) of the tumor, respectively. One tumor in each group were collected on the second day for the H&E staining.

Immune evaluation: When the tumor volumes reached about ~100 mm^3, the mice bearing 4T1 tumor were randomly divided into two groups and received the following treatments: (1) Control, and (2) TiS_X NSs (i.t. injection, 5 mg/kg) (n=5 per group). After the treatments, the mice were sacrificed at the 7th day. The tumors were homogenized to collect the supernatant solution by the centrifugation. Then, the TNF-α, IL-6, and IL-12-P70 were detected by the enzyme-linked immunosorbent assay.

***In vivo* toxicity evaluation**

For long-term toxicity evaluation, the healthy mice were intravenously injected with PEG-TiS_X NSs (10 mg/kg) and sacrificed at different time points (1st, 7th, and 14th, n=5 per time point). The major organs (heart, liver, spleen, lung, kidney, and brain) were collected for both H&E staining, and the Ti ion contents were measured by ICP-OES. Meanwhile, the blood was collected for complete

blood panel analysis and blood biochemistry test. To study the metabolism pathway, mice were kept in the metabolic cages to collect the urine and feces after injection of PEG-TiS_X NSs at various time points, and the contents of Ti ions were also detected by ICP-OES.

Statistic

All quantitative experiments were done in triplicate unless otherwise indicated. Date are presented as mean ± standard deviation (SD). Statistical differences in survival were measured by the log-rank test. The significance was expressed with $*p < 0.05$, $**p < 0.01$, and $***p < 0.001$.

References

[1] a) R. Weber, Z. Riester, L. Hüser, C. Sticht, A. Siebenmorgen, C. Groth, X. Hu, P. Altevogt, J. S. Utikal, V. Umansky, *Journal for ImmunoTherapy of Cancer* **2020**, *8* (2), e000949; b) I. Marigo, E. Bosio, S. Solito, C. Mesa, A. Fernandez, L. Dolcetti, S. Ugel, N. Sonda, S. Bicciato, E. Falisi, F. Calabrese, G. Basso, P. Zanovello, E. Cozzi, S. Mandruzzato, V. Bronte, *Immunity* **2010**, *32* (6), 790.

2. Supporting Figures

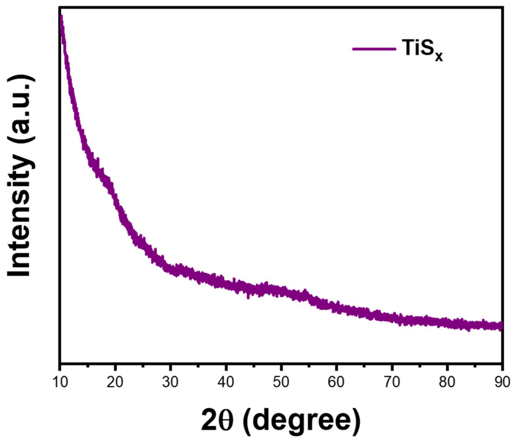

Figure S1. XRD spectrum of TiS_X NSs.

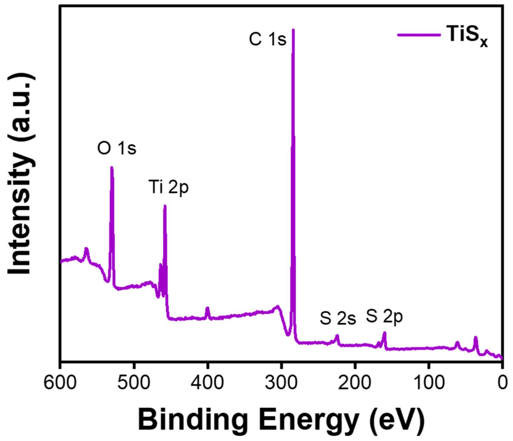

Figure S2. XPS spectra of survey of TiS_X NSs.

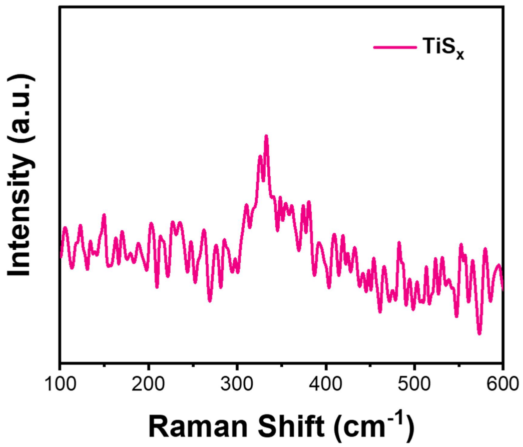

Figure S3. The corresponding Raman spectrum of TiS_X NSs in Figure 1h.

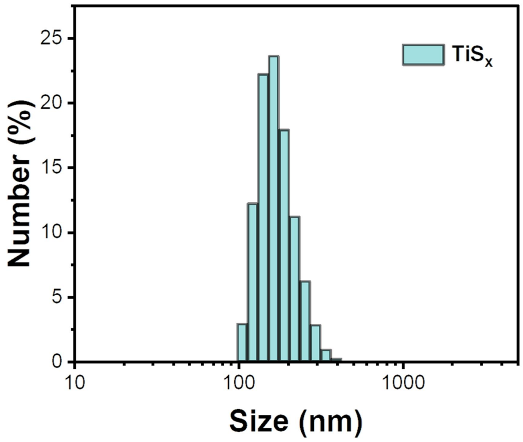

Figure S4. DLS of PEG-TiS_X NSs.

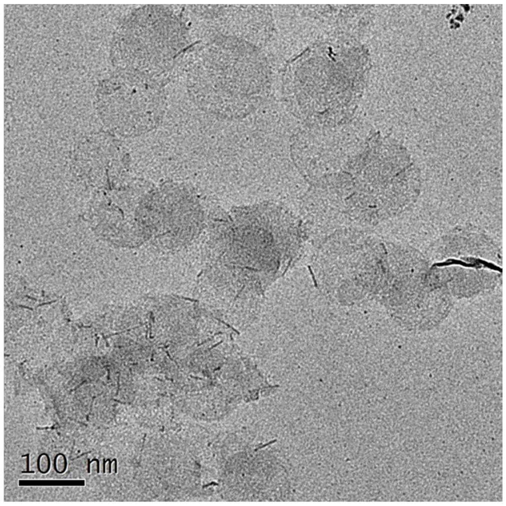

Figure S5. TEM image of PEG-TiS_X NSs.

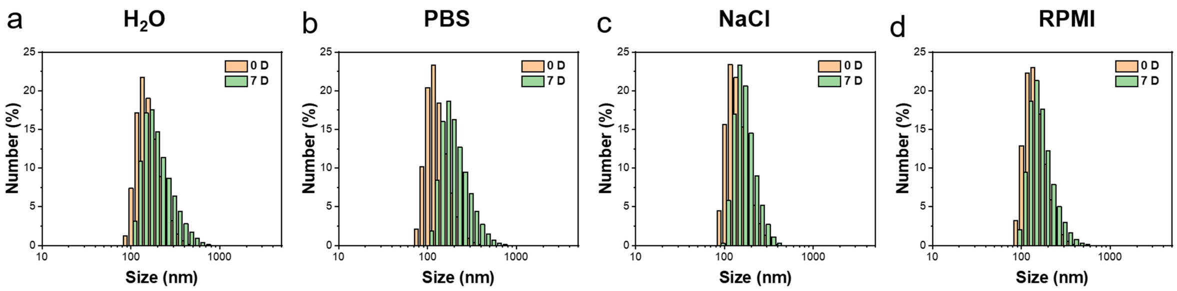

Figure S6. (a-d) DLS of TiSx NSs in H_2O **(a)**, PBS **(b)**, 0.9% NaCl **(c)**, and RPMI **(d)** at 0 D and 7 D, respectively.

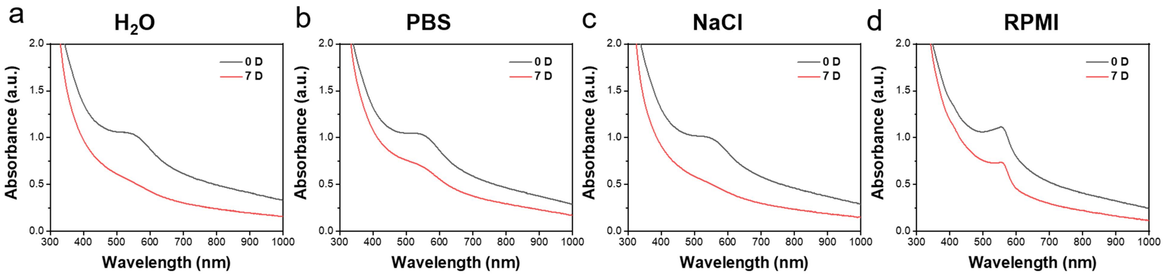

Figure S7. UV-vis-NIR spectra of PEG-TiS_X NSs in H_2O **(a)**, PBS **(b)**, 0.9% NaCl **(c)**, and RPMI **(d)** at 0 D and 7 D, respectively.

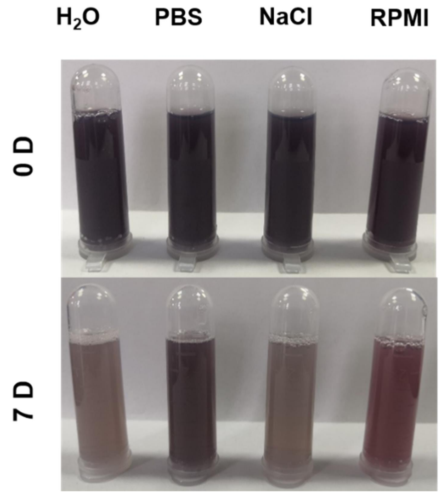

Figure S8. Photograph of PEG-TiS_X NSs in different solutions at 0 D and 7 D, respectively.

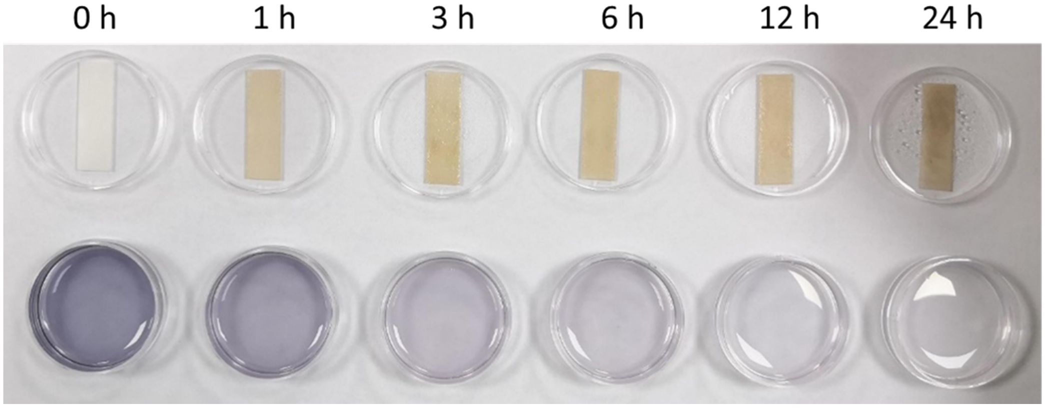

Figure S9. H_2S releasing performance of PEG-TiS_X NSs (50 ppm) with different degradation time using lead acetate test paper as the indicator.

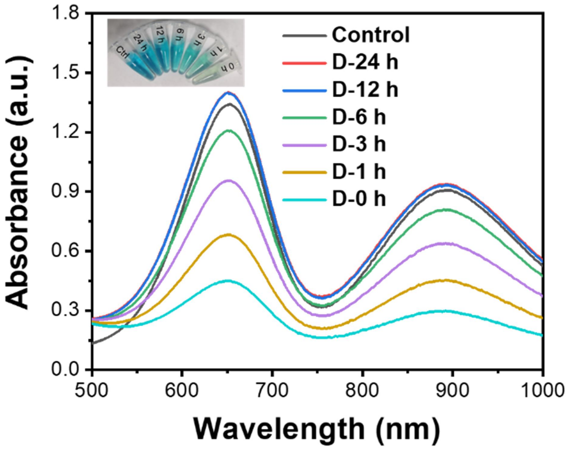

Figure S10. H_2S release performance of PEG-TiS_X NSs (50 ppm) with different degradation time using TMB as the probe.

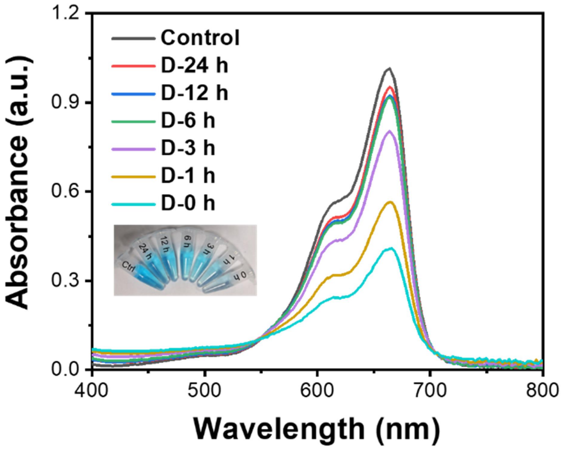

Figure S11. H_2S release performance of PEG-TiS_X NSs (50 ppm) with different degradation time using MB as the probe.

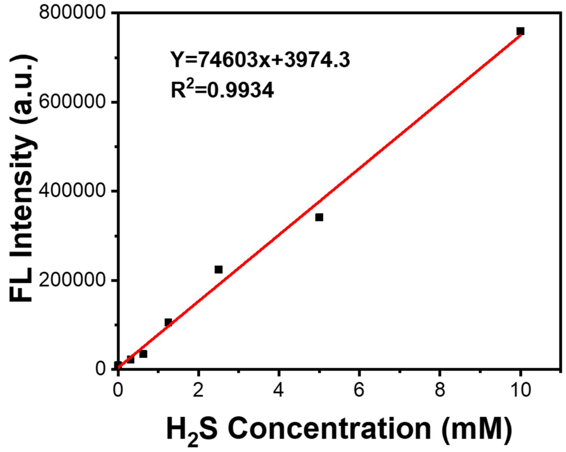

Figure S12. The standard fluorescence curve of Na_2S after co-incubation with WSP-1 probe.

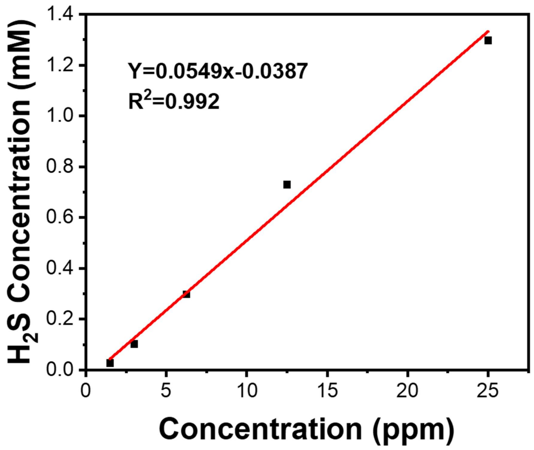

Figure S13. The fluorescence decay curve in Figure 2e.

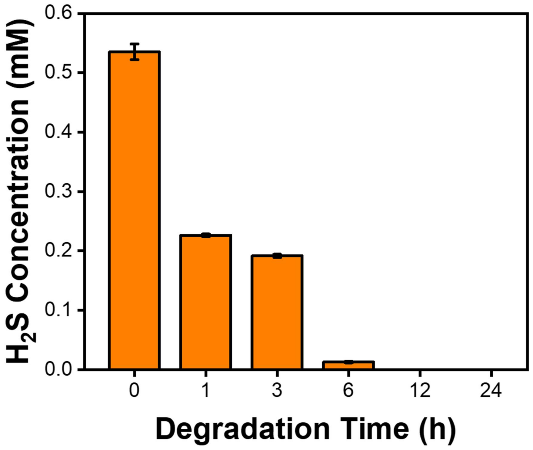

Figure S14. H_2S releasing performance of PEG-TiS_X NSs (10 ppm) with different degradation time using WSP-1 as the probe. Data were presented as mean values ± SD (n=6 biologically independent samples).

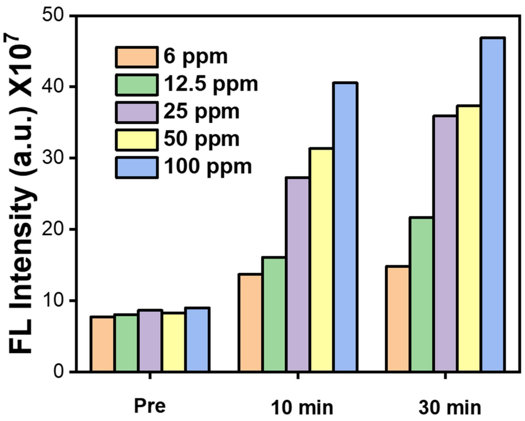

Figure S15. Quantitative analysis of fluorescence signal in Figure 2f.

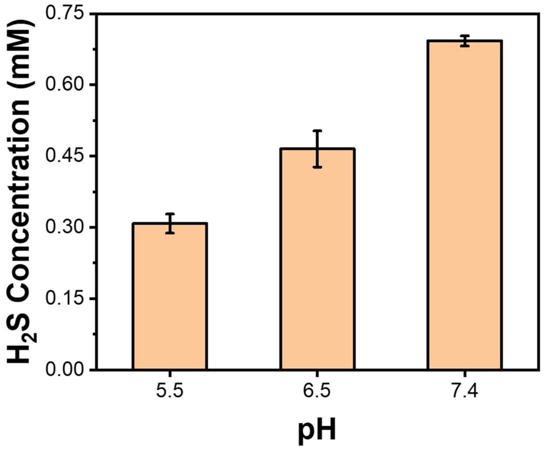

Figure S16. H_2S release performance in different pH. Data were presented as mean values ± SD (n=6 biologically independent samples).

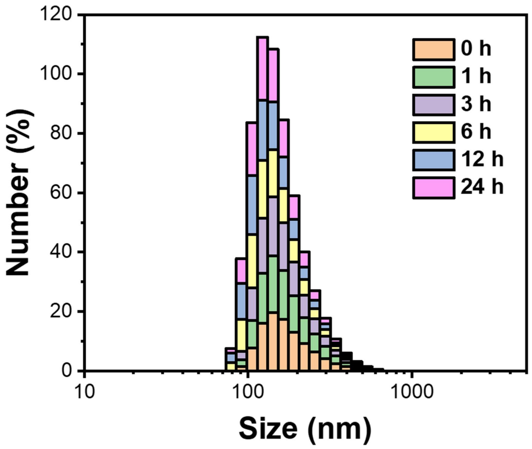

Figure S17. The DLS of PEG-TiS_X NSs at different degradation time.

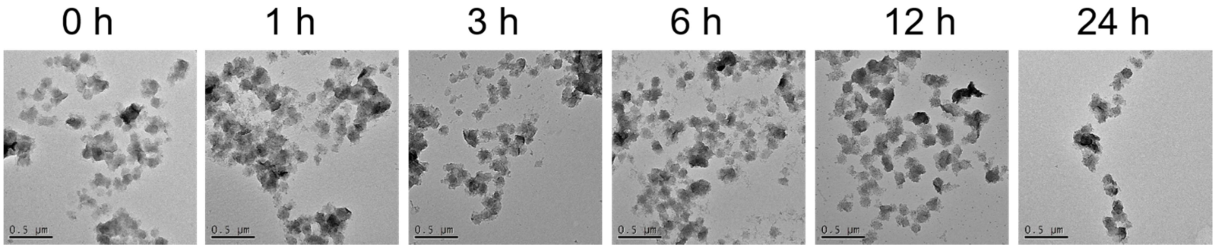

Figure S18. TEM images of PEG-TiS_X NSs at different degradation time.

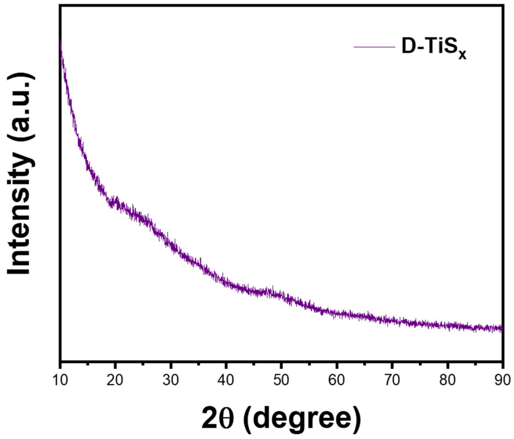

Figure S19. XRD spectrum of D-TiS_X NSs.

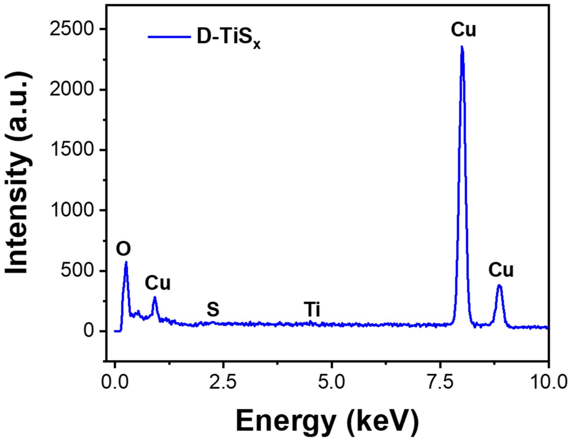

Figure S20. EDX spectrum of D-TiS_X NSs.

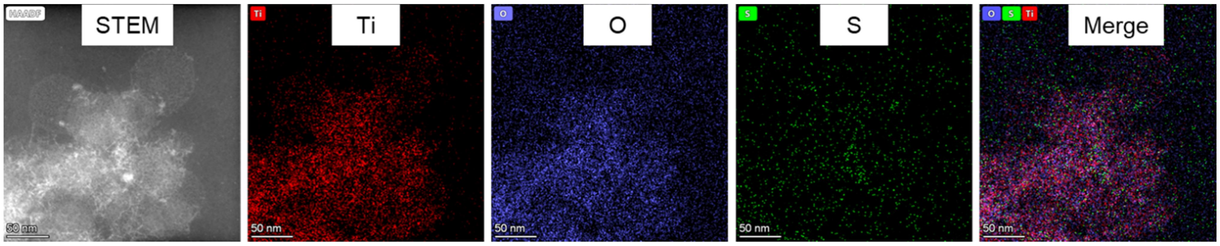

Figure S21. The element mapping images of D-TiS_X NSs.

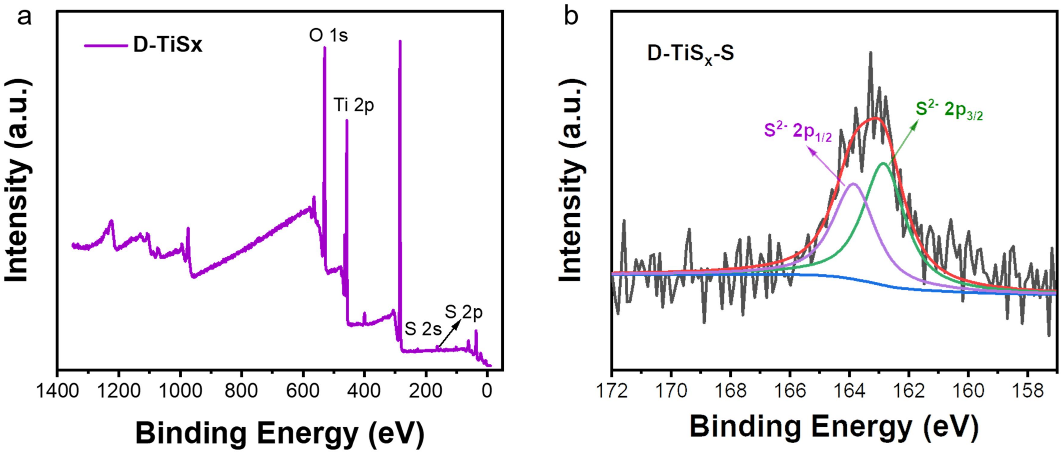

Figure S22. XPS spectra of survey **(a)** and S 2p **(b)** of D-TiS_X NSs, respectively.

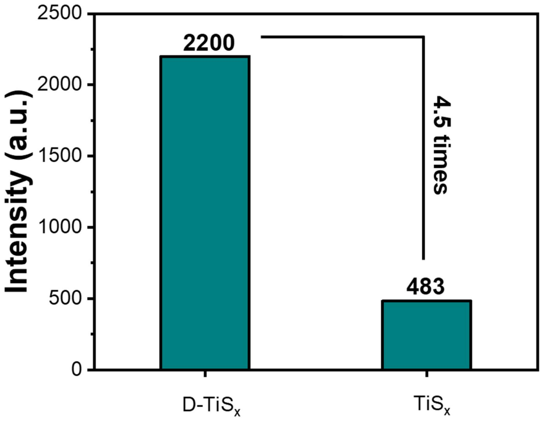

Figure S23. The quantitative comparison of vacancy signal in PEG-TiS_X NSs at different degradation level.

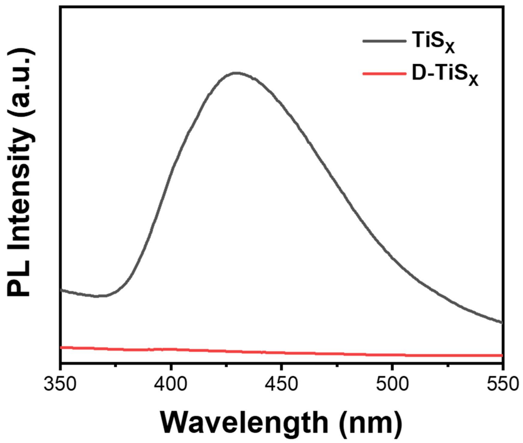

Figure S24. PL spectra of PEG-TiS_X NSs and PEG-D-TiS_X NSs.

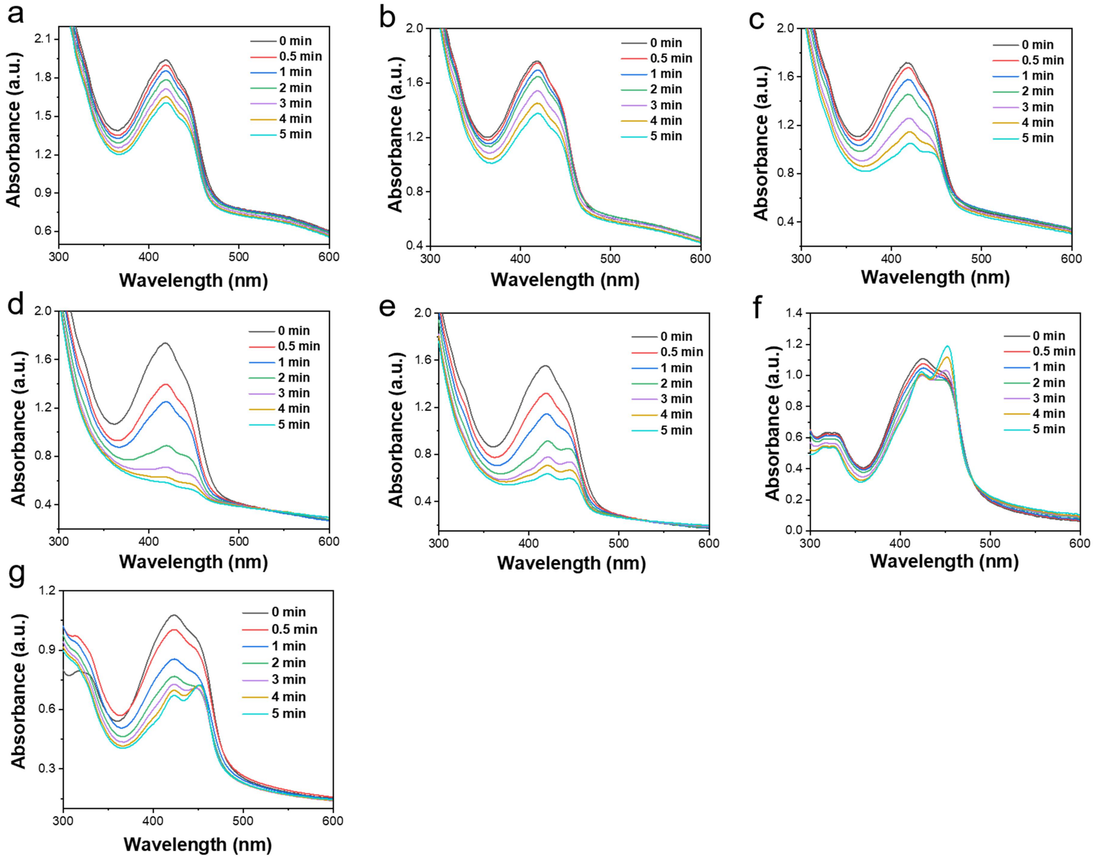

Figure S25. The ROS generation ability of PEG-TiS_X NSs after degradation for 0 h **(a)**, 1 h **(b)**, 3 h **(c)**, 6 h **(d)**, 24 h **(e)** as compared with H_2O **(f)** and commercial TiO_2 **(g)** under US irradiation with DPBF as the probe.

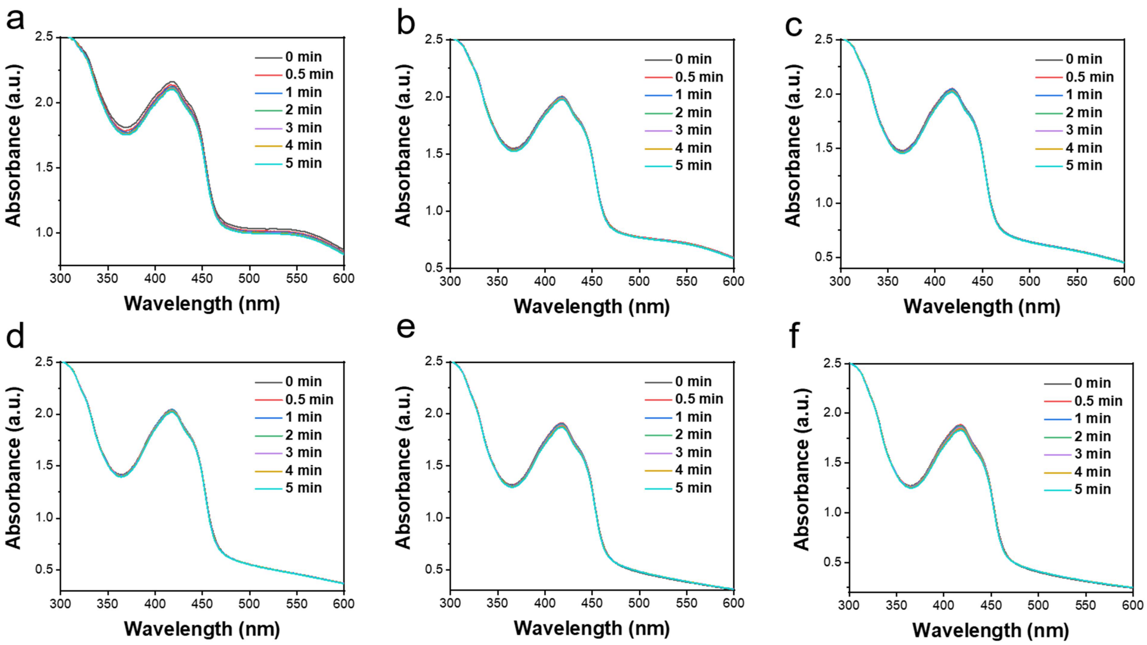

Figure S26. The ROS generation ability of PEG-TiS_X NSs after degradation 0 h **(a)**, 1 h **(b)**, 3 h **(c)**, 6 h **(d)**, 12 h **(e)** as compared with H_2O **(f)** without US irradiation.

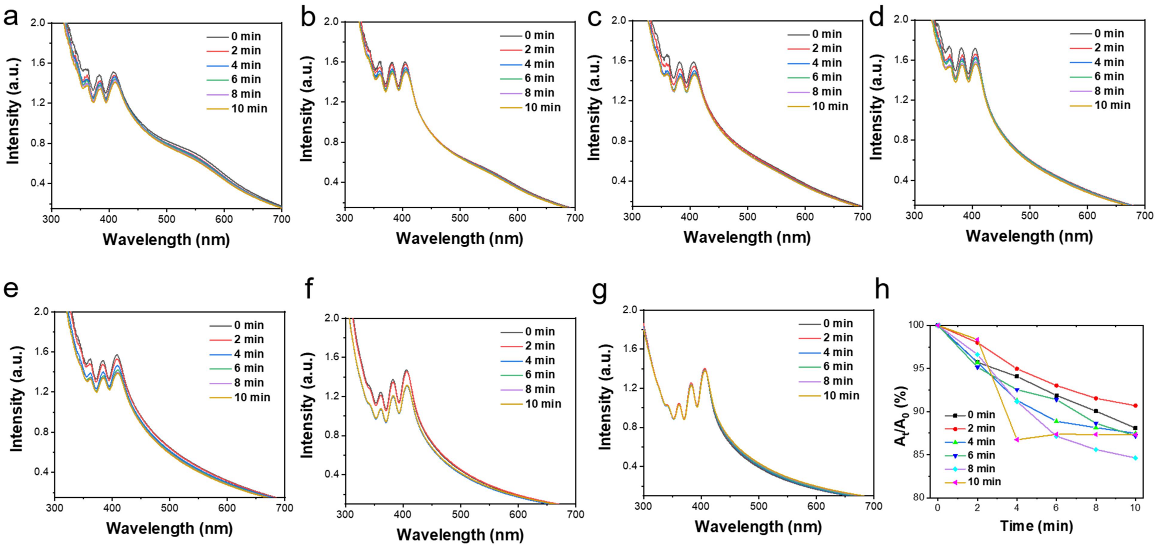

Figure S27. The ROS generation ability of PEG-TiS_X NSs after degradation for 0 h **(a)**, 1 h **(b)**, 3 h **(c)**, 6 h **(d)**, 12 h **(e),** 24 h **(f)**, H_2O **(g)**, and the statistics **(h)** under US irradiation with DPA as the probe.

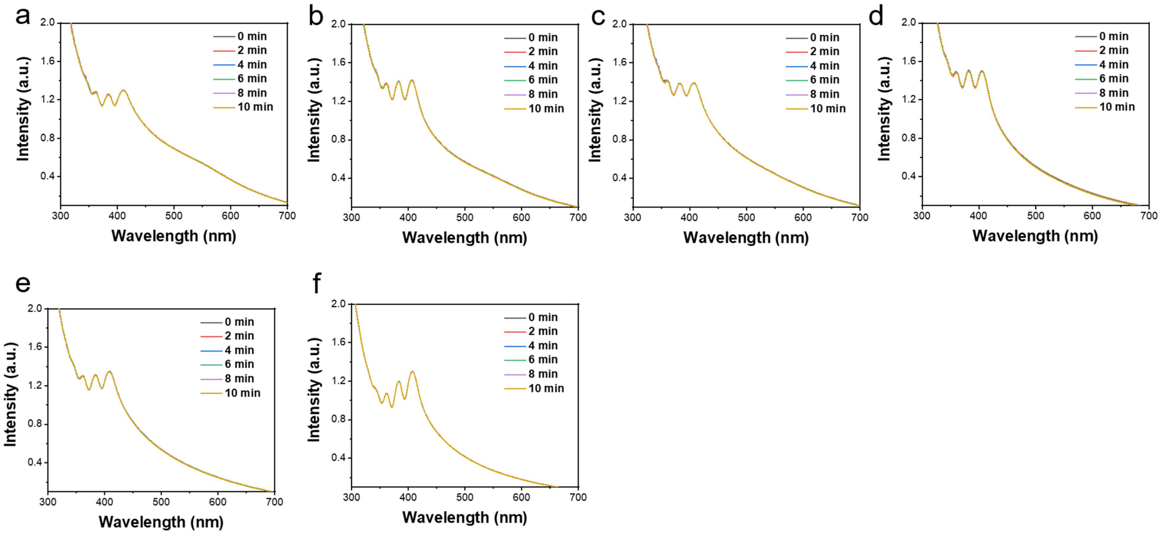

Figure S28. The ROS generation ability of PEG-TiS_X NSs after degradation for 0 h **(a)**, 1 h **(b)**, 3 h **(c)**, 6 h **(d)**, 12 h **(e),** 24 h **(f)** without US irradiation.

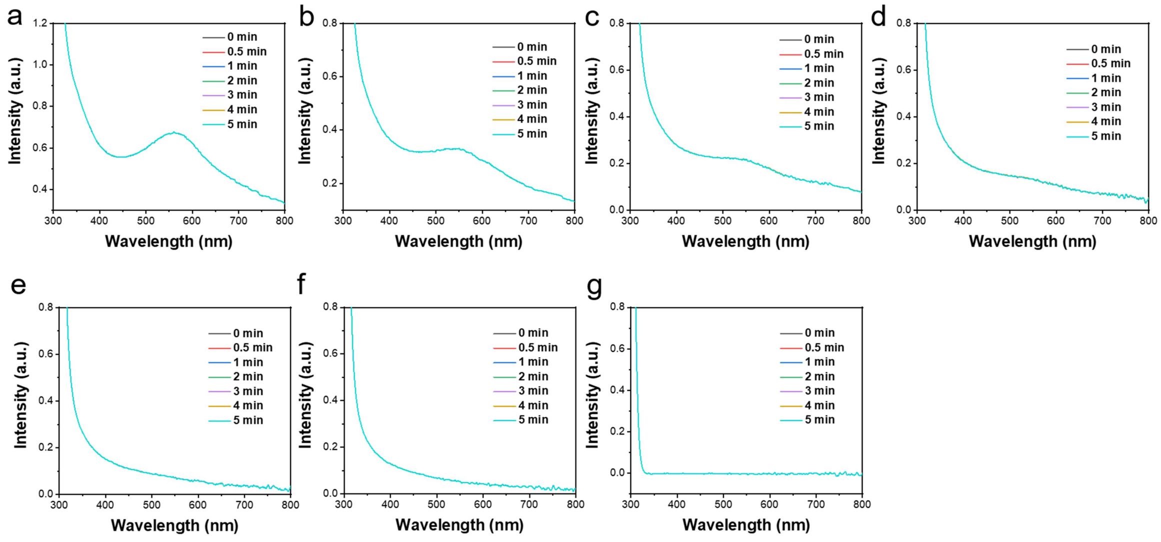

Figure S29. The ROS generation ability of PEG-TiS_X NSs after degradation for 0 h **(a)**, 1 h **(b)**, 3 h **(c)**, 6 h **(d)**, 12 h **(e),** 24 h **(f)**, and H_2O **(g)** under US irradiation with TMB as the probe.

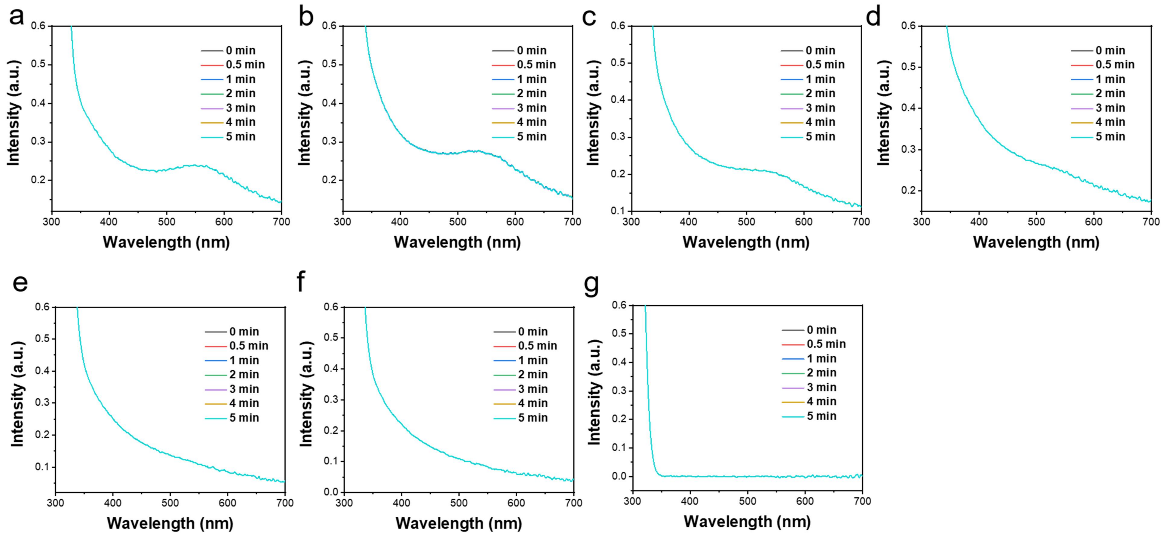

Figure S30. The ROS generation ability of PEG-TiS_X NSs after degradation for 0 h **(a)**, 1 h **(b)**, 3 h **(c)**, 6 h **(d)**, 12 h **(e),** 24 h **(f)**, and H_2O **(g)** under US irradiation with OPD as the probe.

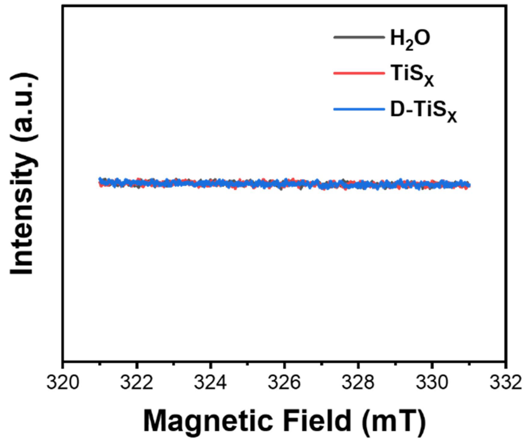

Figure S31. ESR spectra exhibiting ·OH generation.

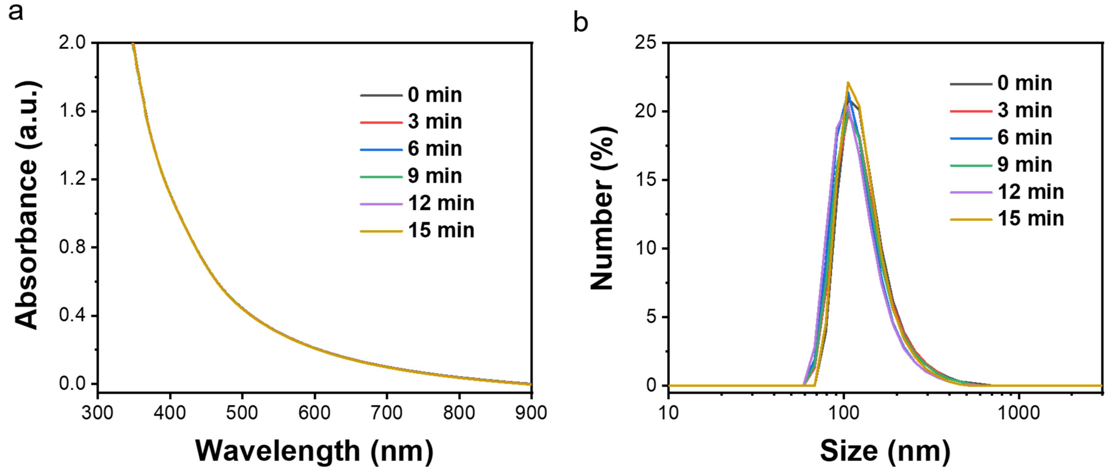

Figure S32. The UV-vis-NIR spectra **(a)** and DLS **(b)** of PEG-D-TiSX NSs after US irradiation at different time points.

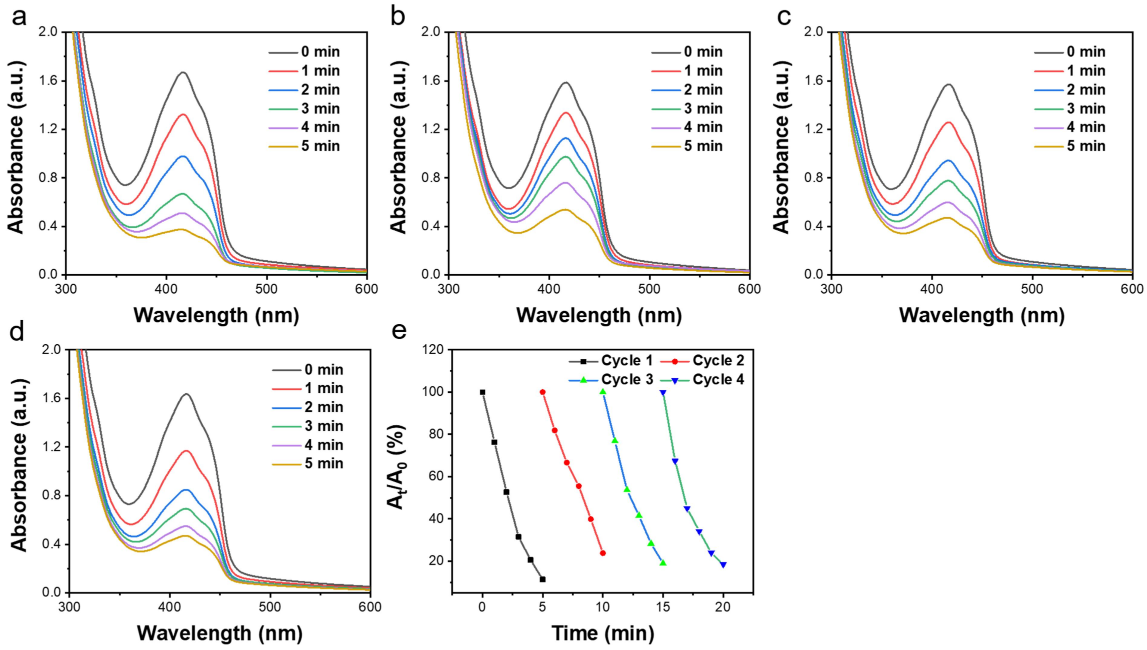

Figure S33. The ROS generation stability of the PEG-D-TiS_X NSs with DPBF under US irradiation in four cycles. Cycle 1 **(a)**, cycle 2 **(b)**, cycle 3 **(c)**, cycle 4 **(d)**, and statistics **(e)**.

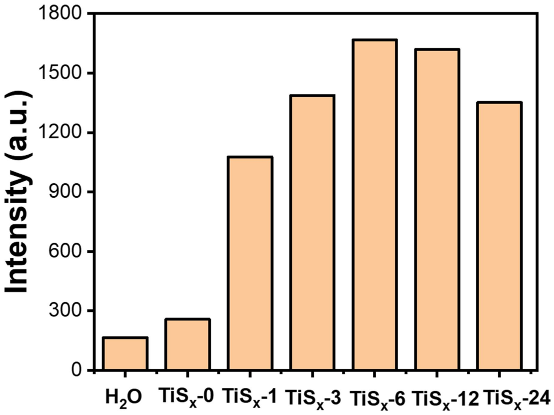

Figure S34. Quantitative analysis of ESR signal in Figure 2m.

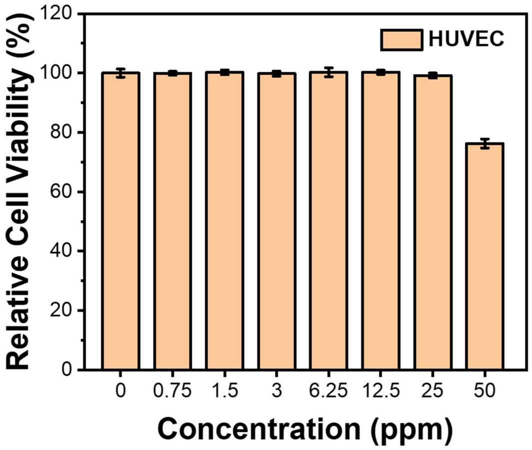

Figure S35. Relative cell viability of HUVECs with the TiS_X NSs with various concentrations for 12 h. Data were presented as mean values ± SD (n=3 biologically independent samples).

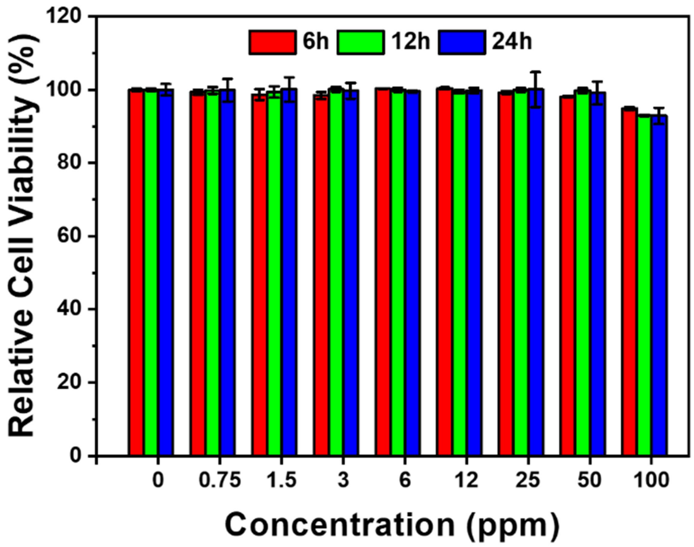

Figure S36. Relative cell viability of 4T1 cells with the PEG-D-TiS_X NSs with various concentrations for 6, 12, and 24 h. Data were presented as mean values ± SD (n=3 biologically independent samples).

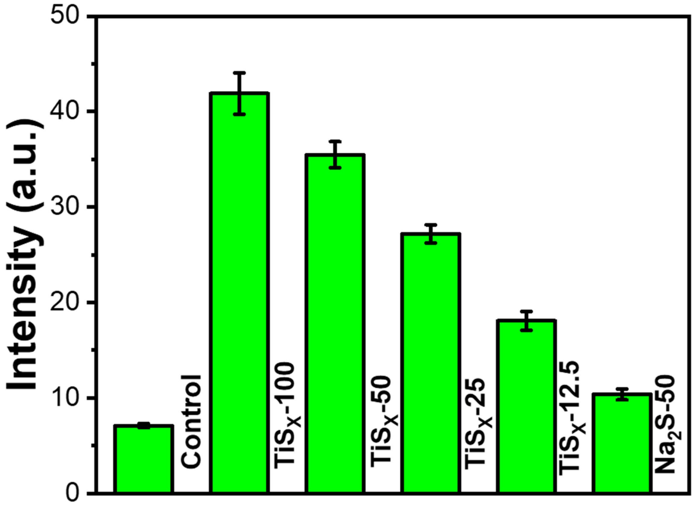

Figure S37. Quantitative analysis of H_2S release in Figure 3e. Data were presented as mean values ± SD (n=6).

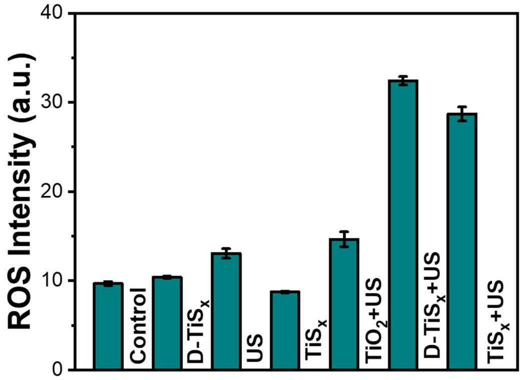

Figure S38. Quantitative analysis of ROS generation in Figure 3h. Data were presented as mean values ± SD (n=6).

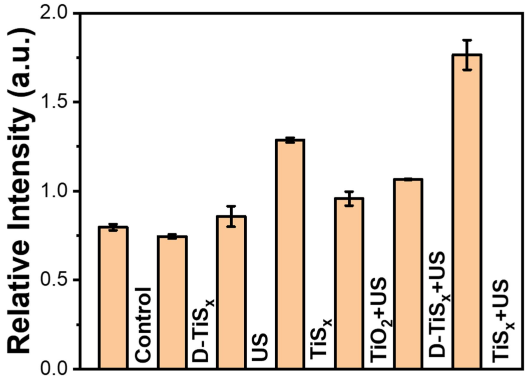

Figure S39. Quantitative analysis of JC-1 signal in Figure 3i.

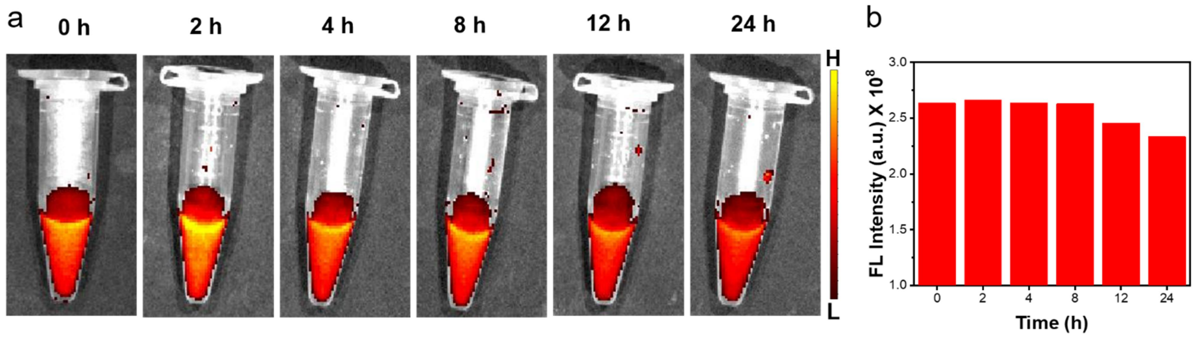

Figure S40. The fluorescence imaging of the Cy5.5-labelled PEG-TiS_X NSs **(a)**, and quantitative

analysis of fluorescence intensity **(b)**.

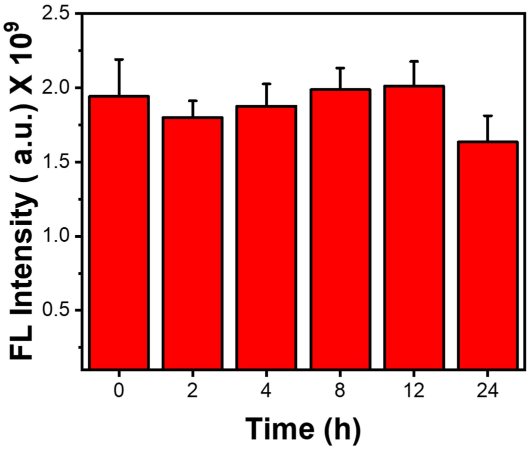

Figure S41. Quantitative analysis of fluorescence intensity in Figure 4b. Data were presented as mean values ± SD (n=3 biologically independent mice).

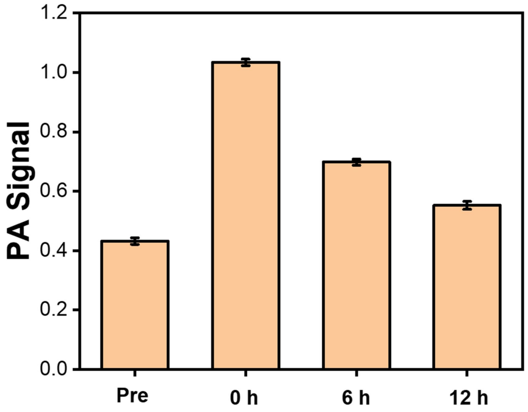

Figure S42. Quantitative analysis of PA intensity in Figure 4c.

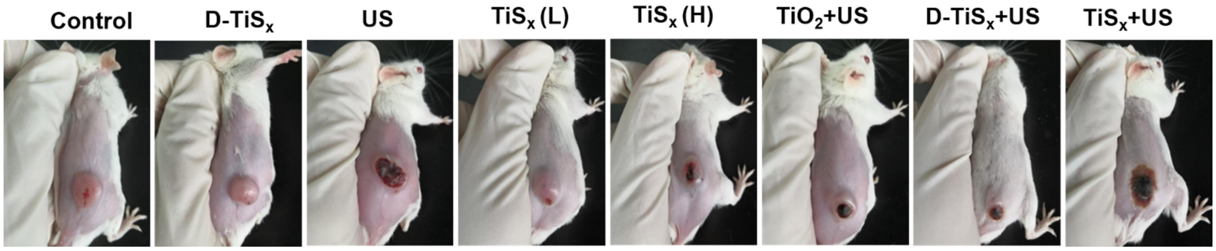

Figure S43. The photograph of mice after various treatments on 6 D.

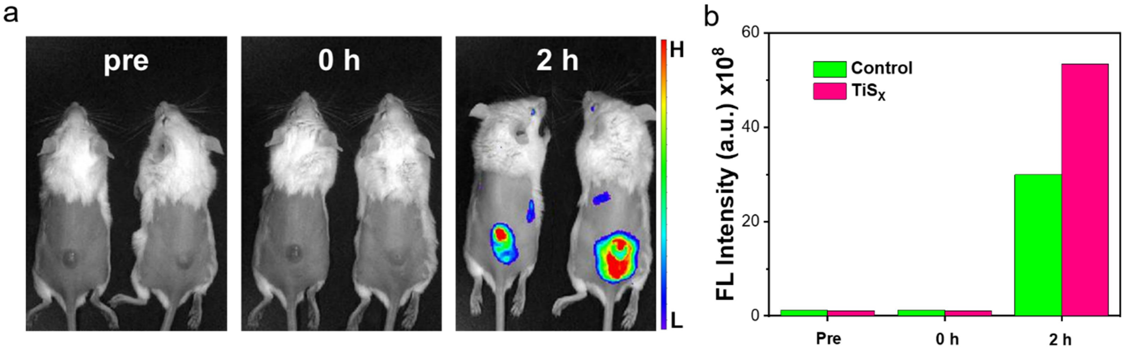

Figure S44. The fluorescence imaging with H_2S probe and PEG-TiS_X NSs in different time **(a),** and quantitative analysis of fluorescence intensity **(b)**.

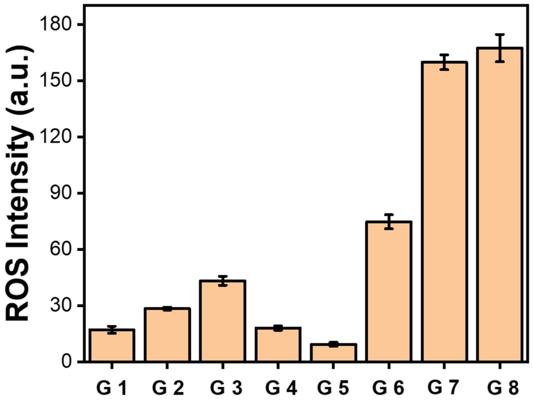

Figure S45. Quantitative analysis of ROS generation in Figure 4h. Data were presented as mean values ± SD (n=6).

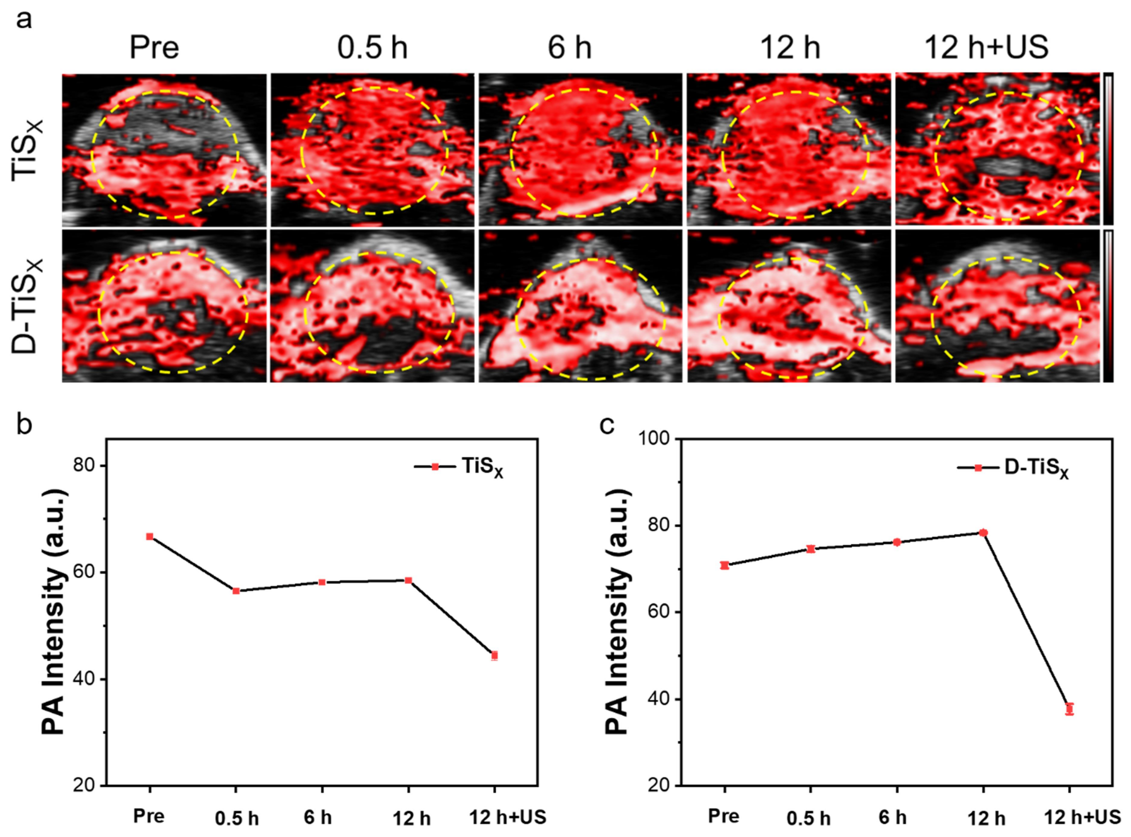

Figure S46. **(a)** The PA imaging of PEG-TiS$_X$ NSs and PEG-D-TiS$_X$ NSs at different time points and with the ultrasound irradiation. **(b&c)** the Quantitative analysis of the PA intensity in image a.

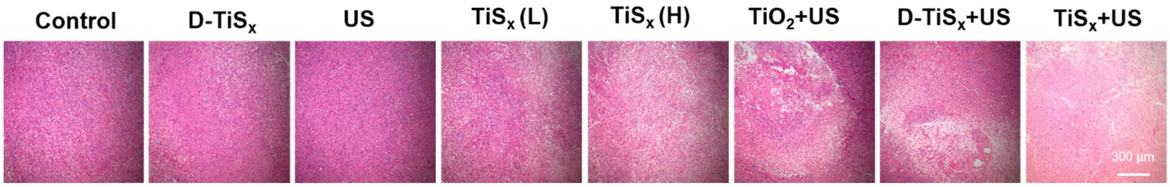

Figure S47. The H&E staining of tumors after different treatments.

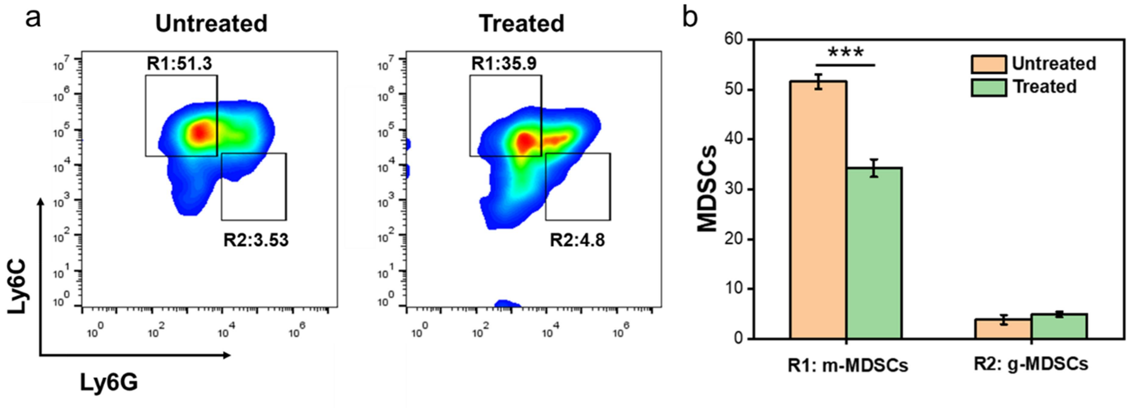

Figure S48. Flow cytometry plots and related quantification of MDSCs **(a&b)** *in vitro* treated with PEG-TiS_X NSs. Data were presented as mean values ± SD (n=3 biologically independent samples).

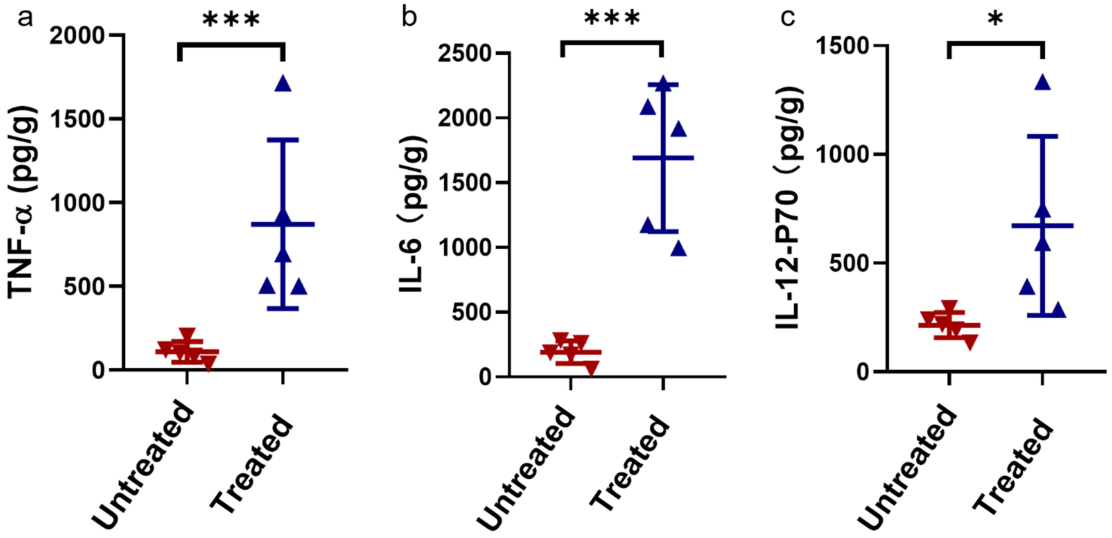

Figure S49. The related factors of TNF- α **(a)**, IL-6 **(b)**, and IL-12-P70 **(c)** in tumors. Data were presented as mean values ± SD (n=5 biologically independent mice).

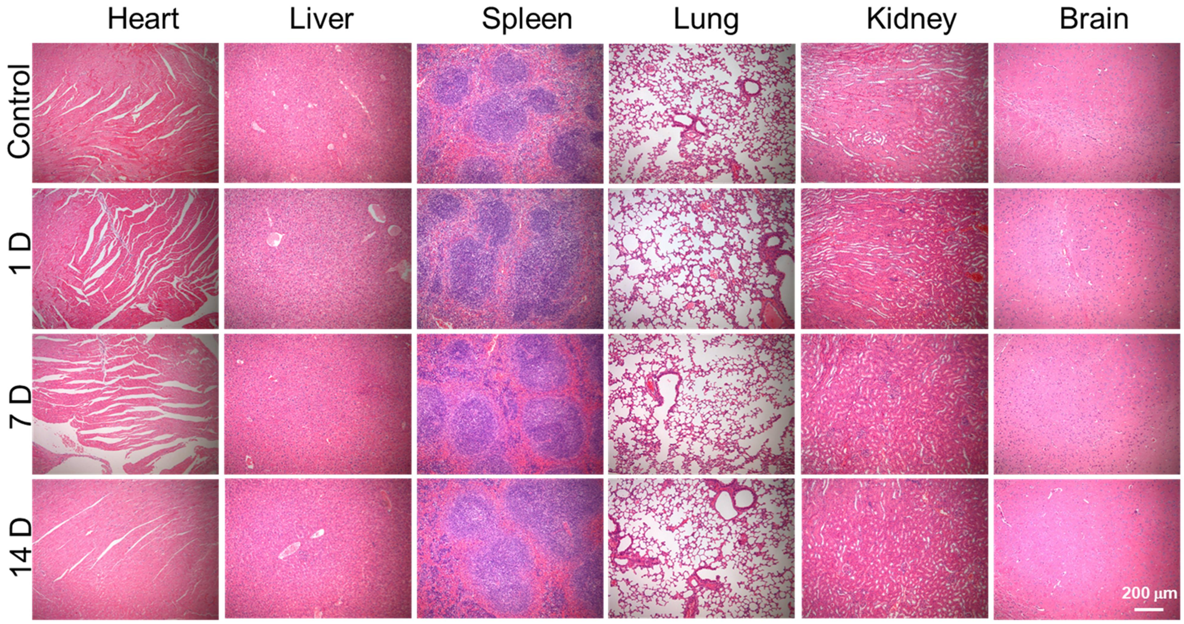

Figure S50. H&E staining of mice major organs before and post i.v. injection with PEG-TiS_X NSs (10 mg/kg) at 1st, 7th and 14th days, respectively.

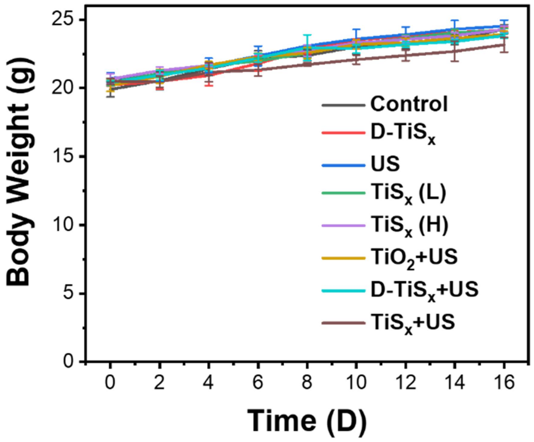

Figure S51. The body weight variation of mice with different treatments. Data were presented as mean values ± SD (n=5 biologically independent mice).

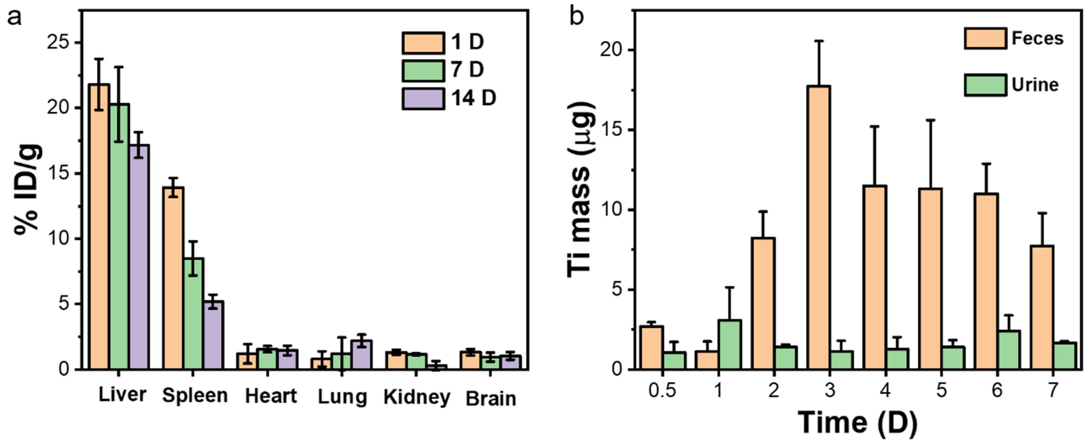

Figure S52. The biodistribution of PEG-TiS_X NSs in different time (**a**) and the Ti mass in feces and urine (**b**). Data were presented as mean values ± SD (n=3 biologically independent mice).

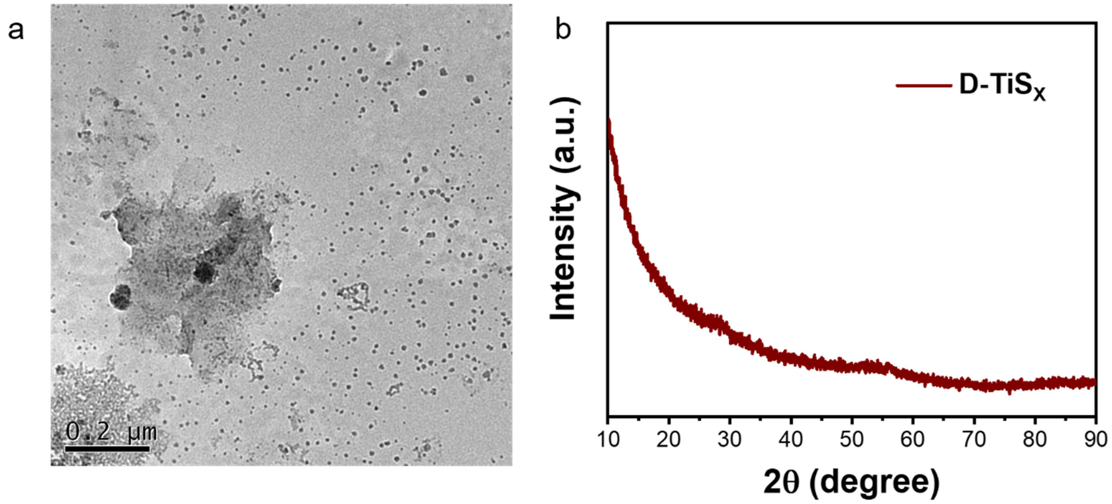

Figure S53. TEM image and XRD spectrum of PEG-TiS_X NSs in 14 D.

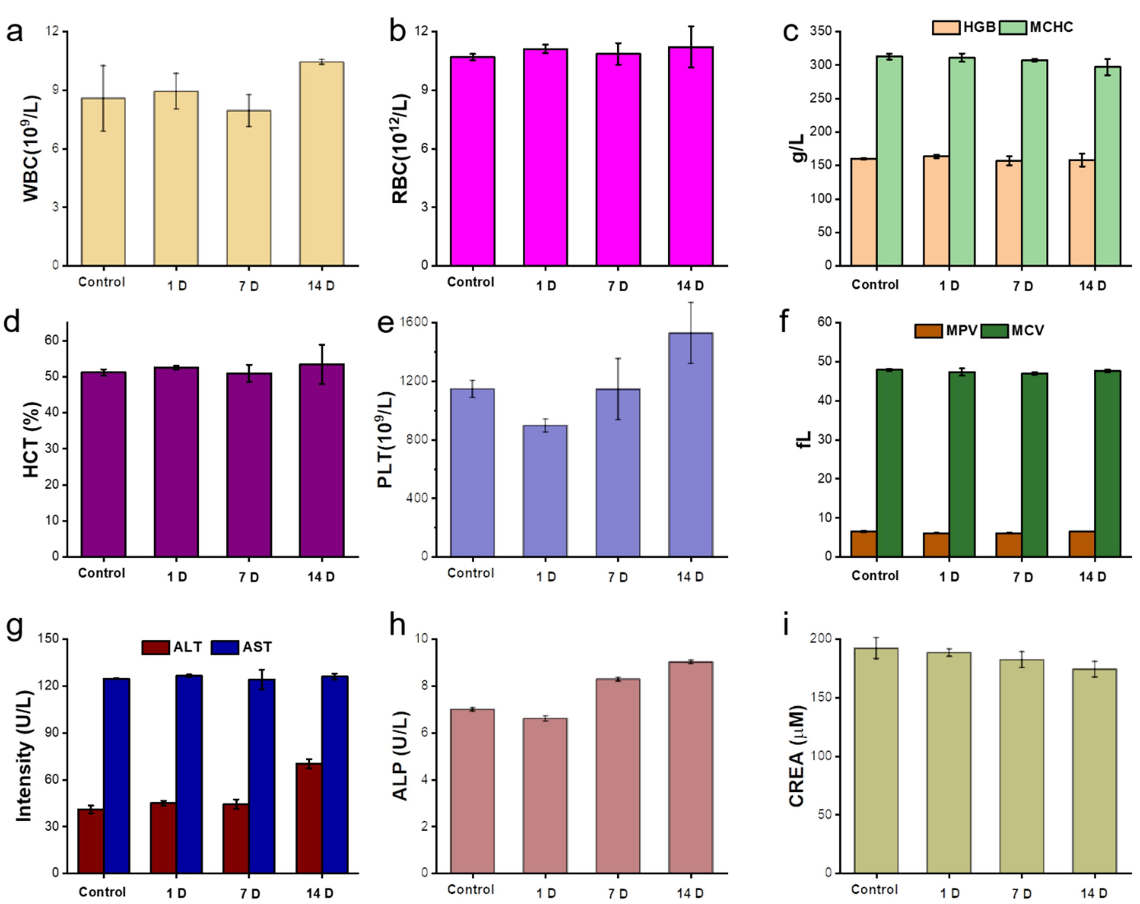

Figure S54. Blood panel analysis (**a-f**) and blood biochemistry test (**g-i**) with healthy Balb/c mice (10 mg/kg). Data were presented as mean values ± SD (n=3 biologically independent mice).
